# Bioarchaeology aids the cultural understanding of six characters in search of their agency (Tarquinia, ninth–seventh century BC, central Italy)

**DOI:** 10.1038/s41598-024-61052-z

**Published:** 2024-05-28

**Authors:** G. Bagnasco, M. Marzullo, C. Cattaneo, L. Biehler-Gomez, D. Mazzarelli, V. Ricciardi, W. Müller, A. Coppa, R. McLaughlin, L. Motta, O. Prato, F. Schmidt, F. Gaveriaux, G. B. Marras, M. A. Millet, R. Madgwick, R. Ballantyne, C.A. Makarewicz, A. Trentacoste, P. Reimer, V. Mattiangeli, D. G. Bradley, C. Malone, C. Esposito, E. M. Breslin, S. Stoddart

**Affiliations:** 1 Dipartimento di Beni Culturali e Ambientali, CRC “Progetto Tarquinia”, Università degli Studi di Milano, Milan, Italy; 2 LABANOF (Laboratorio di Antropologia e Odontologia Forense), Università degli Studi di Milano, Milan, Italy; 3 Institute of Geosciences, Goethe University, Frankfurt, Frankfurt am Main, Germany; 4 Frankfurt Isotope and Element Research Center (FIERCE), Goethe University, Frankfurt, Frankfurt am Main, Germany; 5 Dipartimento di Storia Antropologia Religioni Arte Spettacolo, Sapienza Università di Roma, Rome, Italy; 6 Hamilton Institute, Maynooth University, Maynooth, Ireland; 7 Department of Classical Studies and Program in the Environment, University of Michigan, Ann Arbor, USA; 8 Institute of Archaeology, UCL University College London, London, UK; 9 Magdalene College, Cambridge, UK; 10 Kelsey Museum of Archaeology, University of Michigan, Ann Arbor, USA; 11 School of Earth and Environmental Sciences, Cardiff University, Cardiff, CF10 3AT UK; 12 Cardiff School of History, Archaeology and Religion, Cardiff University, Cardiff, UK; 13 School of Archaeology, University of Oxford, Oxford, UK; 14 Department of Archaeology, University of Cambridge, Cambridge, UK; 15 Institut für Ur- und Frühgeschichte, Christian-Albrechts-Universität zu Kiel, Kiel, Germany; 16 School of Natural and Built Environment, Queen’s University Belfast, Belfast, BT7 1NN UK; 17 Smurfit Institute of Genetics, Trinity College Dublin, Dublin2, Ireland; 18 Dipartimento di Beni Culturali, Alma Mater Studiorum, Università di Bologna, Ravenna, Italy

**Keywords:** Biological techniques, Genetics, Environmental social sciences

## Abstract

Etruria contained one of the great early urban civilisations in the Italian peninsula during the first millennium BC, much studied from a cultural, humanities-based, perspective, but relatively little with scientific data, and rarely in combination. We have addressed the unusual location of twenty inhumations found in the sacred heart of the Etruscan city of Tarquinia, focusing on six of these as illustrative, contrasting with the typical contemporary cremations found in cemeteries on the edge of the city. The cultural evidence suggests that the six skeletons were also distinctive in their ritualization and memorialisation. Focusing on the six, as a representative sample, the scientific evidence of osteoarchaeology, isotopic compositions, and ancient DNA has established that these appear to show mobility, diversity and violence through an integrated bioarchaeological approach. The combination of multiple lines of evidence makes major strides towards a deeper understanding of the role of these extraordinary individuals in the life of the early city of Etruria.

## Introduction

Etruria hosted one of Italy's great early urban civilisations (first millennium BC) in western central Italy^1,2^. It was interconnected to other populations of the Mediterranean, especially the Greeks and the Latins (later Romans). However, the Etruscan culture was set apart within the scenery of the Mediterranean in terms of language and social organisation, especially from the point of view of gender relations, which were not so hierarchical. Etruscan ways challenged the traditional norms of the classical world and were considered alternative in classical written sources. Direct Etruscan written sources are lost and this is probably one reason for the relatively little scholarly attention applied to this culture outside Italy where archaeological excavations take place and support research.

**Figure 1:**
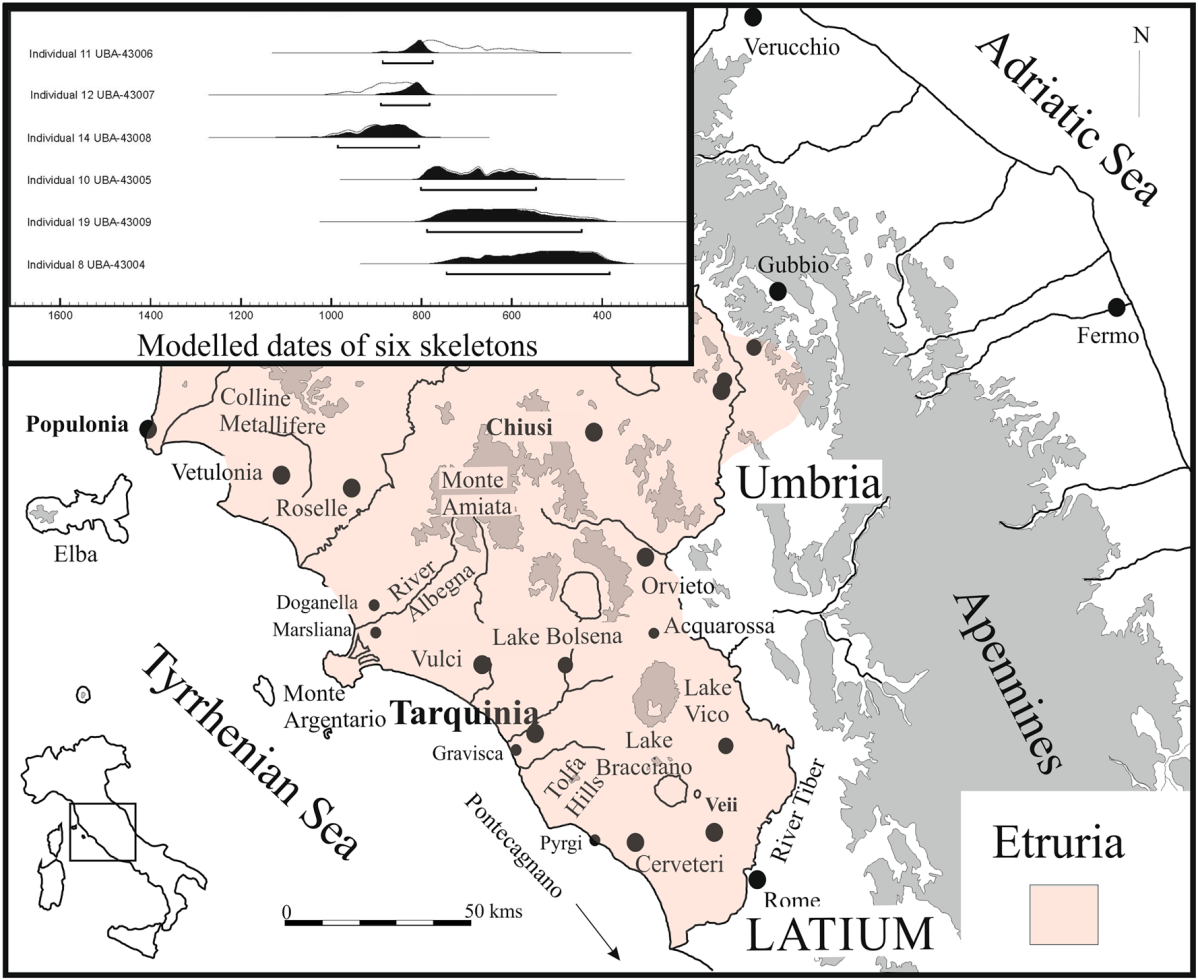
Time and Place. Map of Central Italy showing location of Tarquinia and modelled dates of six skeletons under study.

**Figure 2:**
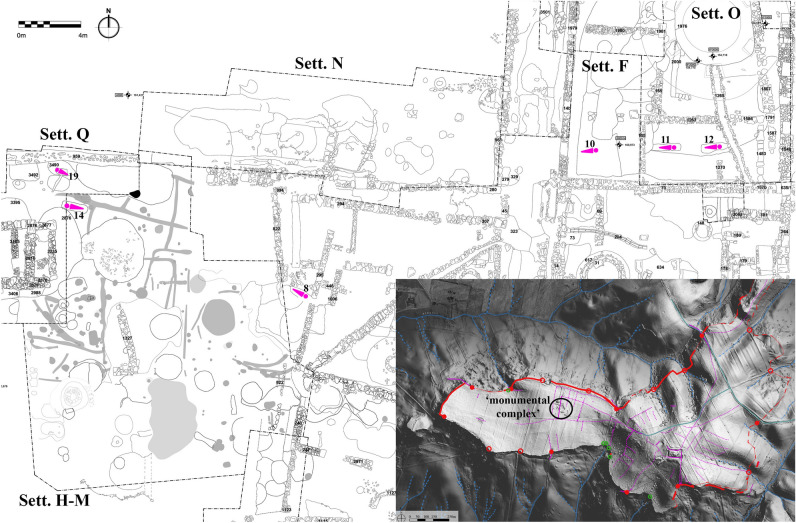
The location of the six skeletons within the ‘monumental complex’ of Tarquinia. Inset shows the position of the monumental complex within the site of Tarquinia, whose walls and gates are indicated in red.

The ancient city of Tarquinia was one of the most important coastal cities of Etruria, alongside Cerveteri to the south and Vulci to the north. Tarquinia rests on the Civita plateau, about 7 km from the Tyrrhenian Sea, and its territory neatly occupies the Marta catchment which drains the volcanic lake of Bolsena^3^ (Fig. 1). The article combines cultural and scientific evidence to answer key archaeological questions, seeking inspiration from the Italian author Pirandello^4^ for its title. It is an explicit attempt to bring into agreement the two worlds of CP Snow^5^; thus, we have endeavoured to match the focus on the unique in the humanistic tradition (and the six skeletons) with the statistical approach typical of the scientific tradition. This is particularly important for pre-Roman Etruscan culture where such approaches are not common (Fig. 2).

The inhabited area of the Civita has been extensively investigated in two places^6–8^, namely the ‘monumental complex’ (*complesso monumentale*) and the Ara della Regina sanctuary^9,10^. The first is one of the deepest stratigraphies so far researched in the city, or indeed in Etruria, with occupation from the tenth century BC to the Roman imperial period, and is the focus of our contribution (see Supplementary Notes). During the course of excavations over the last thirty years, twenty skeletons were recovered, starting at the end of the Bronze Age (tenth century BC).

These discoveries were unexpected, since the twenty individuals were clearly inhumed, almost always in anatomical order, within a sacred area within the city limits and memorialised through a system of signs that kept emerging over the centuries, as shown by the stratigraphy. This contrasts with the major cemeteries that encircle the city. This striking archaeological evidence is the stimulation to explore in this article the exceptional nature of a sample of six representative skeletons from the twenty by asking a series of cultural questions with scientific as well as archaeological evidence:

- Why were these individuals buried in a sacred area of the city?
- Why did all the skeletons share the same practice of inhumation and memorialisation? Were there other characteristics that they held in common?
- Did all these individuals have a common ancestry with other Etruscans communities?
- Were these people born locally or elsewhere?

Our focus is on obtaining insights from a cultural and historical point of view, based on the archaeological and other scientific evidence we have gathered so far. The samples have been collected with these cultural questions in mind. We use the osteobiography of these individuals in a microhistorical perspective, which aims to shed light on the historical understanding of human societies through the particular history of the individuals^11,12^ specifically their unusual treatment by inhumation rather than by cremation and their rare spatial setting. Moreover, the concept of microhistory has more recently been introduced into the field of bioanthropology^13,14^ and there we achieve the same conceptual point of view by combining bioarchaeology and osteobiography. As stated above, our approach is directly focused on integrated research combining the humanistic philological tradition with the statistical analysis of the scientific tradition. We share the same objective of reconstructing the lost profile of Tarquinia from multifaceted different categories of evidence without the constraints imposed by information from classical written sources. Our work proceeds from the analysis of the data to the search for recurring associations between different categories of evidence to draw on a consistent series of results that in the first instance allow for microhistorical reconstructions, and then tap into the overall history of the settlement and finally its connections to macro-history^15^.

The research has five interlinked facets:

- The detailed osteological analysis, based on forensic science and palaeopathology.
- The construction of a solid timeline based on radiocarbon dating integrated with the chronological sequence obtained through archaeological investigation (stratigraphy and archaeological artefacts) and merged within Bayesian statistical models. This is crucial to bridge the gap between chronologies obtained through traditional sources and those through physico-chemical analyses. This approach has significance both for Etruria, where phasing has often been constructed by typological sequences and cross-dating, and for Europe, by contributing to an improved understanding of the Hallstatt calibration curve plateau which affects the period between c. 800 and 400 BC^16^. The presence of secure dating greatly enhances the interpretation of our other data sources.
- Multi-isotope analysis of carbon (δ^13^C), nitrogen (δ^15^N), oxygen (δ^18^O) and strontium (^87^Sr/^86^Sr).
- The construction of a local strontium isotope (^87^Sr/^86^Sr) reference set for the area of Tarquinia, with which the values of our human remains can be compared. Our reference set has focused on sediment (soil) samples, plants, archaeological fauna, taken from the Civita plateau and cremated young and older children (1–10 years of age) from the nearby Iron Age necropolis of Villa Bruschi Falgari.
- The successful implementation of ancient DNA (aDNA) on five of the six human remains, set against open-source data from contemporary central Italian populations which have been previously published^17,18^.

## Results

A summary of the chronological, spatial, osteological, δ^13^C, δ^15^N, ^87^Sr/^86^Sr, δ^18^O, ^14^C and aDNA analyses is reported in Table 1. Other more detailed information for each analyses is reported in the Supplementary Tables.

**Table 1:** Main results from this research.

Skeleton number	US	Box	Sect	General anthropological info	Genetic sex	preliminary ^14^C results	Spatial position	Chron. from archaeol context	^87^Sr/^86^Sr	(± 95% c.l.)	δ^18^O_ca_ V-SMOW(‰)	Carbon/Nitrogen results
Individual 8	1274	C795	M	Middle aged female adult, inhumation. 160 cm high	F	660–400	To W of the natural cavity below a wall	680–630 BC	0.711150	0.000031	25.07	65% fish diet
Individual 10	545/1	/	F	Middle aged male adult, inhumation. 172 cm high. Relatively tall	M	790–580	To north of original cavity. Fragment of anchor above in stratigraphy. Greek bowl fragment	750–725 BC	0.708878	0.000024	27.60	Foods from the land (25% fish diet)
Individual 11	1601	C990	O	Old Female adult, inhumation	F	830–790	A little further east than 10 in hut. Fibulae. Claimed structured deposition of ash on top and pits	810–750 BC	0.712534	0.000028	24.45	Fish diet & foods from the land (50% fish diet)
Individual 12	1602	C991	O	Young female adult, inhumation	F	840–790	A little further east than 10 in hut. Fibulae	810–750 BC	0.708922	0.000023	26.12	Foods from the land (20% fish diet)
Individual 14	2924	C1695	HM	Middle aged female, inhumation	F	920–830	In another eliptical structure, probably a hut	9th-7th cent BC	0.708955	0.000029	28.62	Foods from the land (25% fish diet)
Individual 19	3493	C2126	Q	Old Male adult inhumation. 167 cm high	M	740–550	Crouched burial. Near another eliptical structure, probably a hut	9th-7th cent BC	0.708992	0.000031	25.90	Fish diet (70% fish diet)

### Multi isotope analysis

#### Carbon and nitrogen isotopes

**Figure 3:**
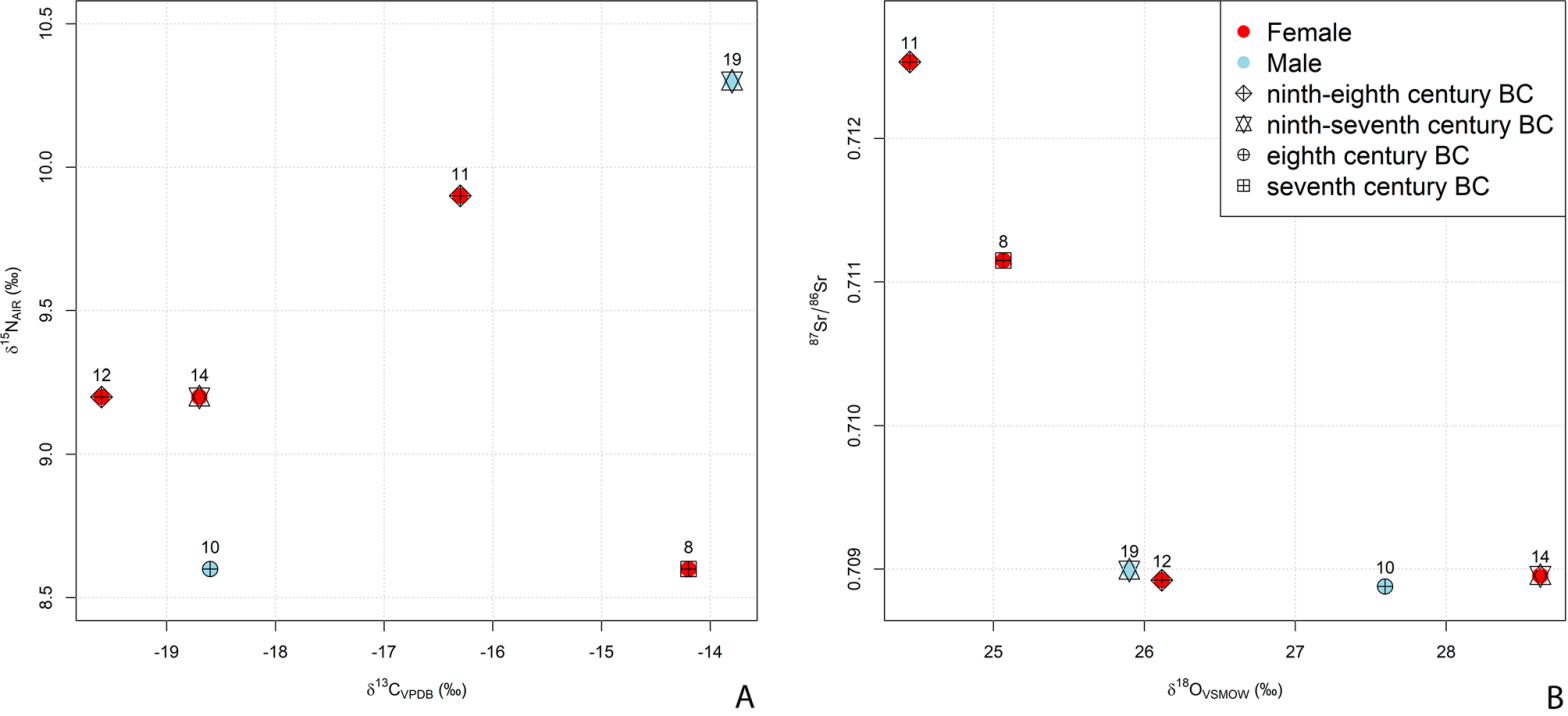
Scatter plots with (A) carbon (δ^13^C) and nitrogen (δ^15^N) and (B) strontium (^87^Sr/^86^Sr) and oxygen (δ^18^O) values of the six individuals from Tarquinia Civita divided for sex and different chronologies.

All the six samples met quality control criteria (Supplementary Table S2). C:N ratios fall in the range between 2.9 and 3.6, indicating good collagen preservation^19^. The δ^15^N values range from 8.6‰ to 10.3‰ and δ^13^C ranges from −19.6‰ to −13.8‰. The standard deviation (SD) for δ^13^C is 0.7‰ and SD for δ^15^N is 2.5‰ (Fig. 3A).

#### Distribution of biologically available strontium (BASr) at Tarquinia

The distribution of strontium isotopes across the landscape is strongly influenced by geology^20^ and further modified by soil formation processes, deposition of aeolian dusts, and seaspray effect. Here we systematically sampled plants and soil from lithological units across the Tarquinia landscape to establish a locally referenced bioavailable strontium isotope dataset. The lithologies of this territory originate from the marine transgression that affected the Central Italian region over the Plio-Pleistocene (5–0.8 Ma)^21–23^. Specifically, the site of Tarquinia Civita sits atop a plateau-like hill formed after the erosion of the poorly cemented *Pian della Regina Unit* (Table S3, sample n. 7), composed of mollusca-rich clayey sands, and the consequent exposure of the more competent *Macco Unit* tabular beds is constituted of alternating strata of calcarenite, limestone, and sandy mudstone^21,22^ (Table S3, samples n. 8, 9, 11). To the east these are overlain by fossiliferous clay of the *Macchia della Turchina Unit (Table S3, sample n. 13).* The surrounding slopes, topographically and stratigraphically beneath the *Macco Unit,* are composed of thick deep basinal muddy units belonging to the *Fosso San Savino Unit*^24^ (Table S3, sample n. 15). According to the recently built isoscape map by Lugli and colleagues^25^, the ^87^Sr/^86^Sr for this area ranges between 0.7091 and 0.7094 (Supplementary Figure S1), within a 5 km radius. Further information on the geology of Tarquinia is available in the Supplementary Notes.

All the ^87^Sr/^86^Sr results are reported in Table S3. The Tarquinia samples used for the local reference (archaeological fauna from the site of Tarquinia Civita: pigs n = 4, hare n = 1, sheep/goat n = 1; modern seeds n = 5; soil samples n = 5; Tarquinia Villa Bruschi older children (OC) n = 4 and young children (YC) n = 6) show ^87^Sr/^86^Sr values in the range of 0.7090–0.7102 (Supplementary Figure S4). Young children (YC) and older children (OC) from Tarquinia Villa Bruschi Falgari show a range of (0.7091–0.7095) with YC having slightly broader values. Samples of soil and seeds from modern plants are in overall good agreement (0.7090–0.7092; 0.7091–0.7094 respectively), with seeds showing slightly more radiogenic values. All faunal samples were contemporary to the skeletons, except for the hare which was from the first centuries BC. Fauna samples have a range of 0.7090–0.7102, with two pigs (Tq-Bs-17 = 0.7099; Tq-Bs-19 = 0.7102), which may have derived from, or eaten human food discard from, elsewhere, since they had the most radiogenic values. The hare sample also yields slightly higher values compared with the other samples (Tq-Bs-21 = 0.7094). If we exclude the two pigs (Tq-Bs-17 and Tq-Bs-19), the faunal strontium isotopic values for Tarquinia range from 0.7090 to 0.7094 (Supplementary Figure S4). YC and OC fit with the other values of the reference data set and are slightly higher than the values for Tarquinia detected in the isoscape^25^. Finally, a conservative estimate of the BASr of all these data suggests that the range 0.7090–0.7095 characterises the local area of Tarquinia.

#### Individual mobility of the six individuals: oxygen and strontium isotopes values

Oxygen, carbon and strontium isotope results for Tarquinia Civita are reported in Supplementary Table S3. The samples analysed were second permanent molars (M2; n = 5), except for Individual 8 where an M1 was sampled since the M2 was not available (for more information see Material and Methods below). A bulk chunk sample for strontium isotope analyses was cut mid-tooth crown horizontal to the tooth growth axis and a bulk sample for oxygen isotope analyses drilled alongside. Samples for oxygen and strontium were taken from the same segment of the tooth (see Supplementary Notes). δ^18^O values measured from the enamel carbonate fraction was converted into the V-SMOW scale (V-SMOW = 1.0309 × δ^18^O_VPDB_ + 30.91^26^) (Fig. 3B).

The ^87^Sr/^86^Sr ratios for Tarquinia human samples range between 0.70888 and 0.71253 (Supplementary Table S3; mean = 0.70991 ± 0.00156), which is larger than BASr. Two individuals (8 and 11) are clear-cut outliers (0.71115 and 0.71253, respectively) and fall far outside the local bioavailable strontium (0.7090–0.7095). The other four individuals exhibit remarkably similar strontium isotopic values ranging from 0.70888 to 0.70899 (Fig. 3B).

### Ancient DNA

The initial summary of the six Tarquinian individuals (reduced to five with sufficient levels of endogenous DNA) and their broad sequencing results, contamination estimates, and uniparental markers, can be found in Supplementary Table S4.

#### Uniparental markers

##### Y-chromosome Haplogroups

Both males in Tarquinia, Individual 10 and Individual 19, are assigned to the J2b/J-M12 lineage (Table 1 and Supplementary Table 9). On the basis of covered positions, Individual 10 is assigned to J-M12/J2b precisely, and Individual 19 to the J-M241/J2b2a haplogroup. As the male samples were sequenced to a depth of 0.86 and 1.7X genomic coverage there was low coverage over most informative Y-chromosome haplogroup markers, and some were missing. For this reason, the possibility of the individuals belonging to a slightly more derived haplogroup cannot be excluded. J2b and its sub-lineages have a present-day geographic distribution from South Asia to Europe, and has been hypothesised to have spread to Central Europe via the Balkans around the early Bronze Age^27,28^. J2b and J2b2a (and sub-lineages) have been reported in ancient individuals from across the Mediterranean and central Europe from the Bronze Age, including Iron Age and Medieval individuals from central and south Italy. Amongst these individuals are R474, an Etruscan individual from the site of Civitavecchia, and CSN004, an undated putatively Etruscan individual from Campiglia dei Foci^17,18^.

##### Mitochondrial haplogroups

All five individuals carry mitochondrial haplogroups typical of post-Neolithic Europe (Table 1 and Supplementary Table S8). None of the five individuals share a common mitochondrial haplogroup.

#### Kinship analysis

No close autosomal kinship was detected up to the 4th degree was detected between any pair of the new individuals, using multiple methods^29,30^. Details can be found in the Supplementary Notes.

#### Principal component analysis

**Figure 4:**
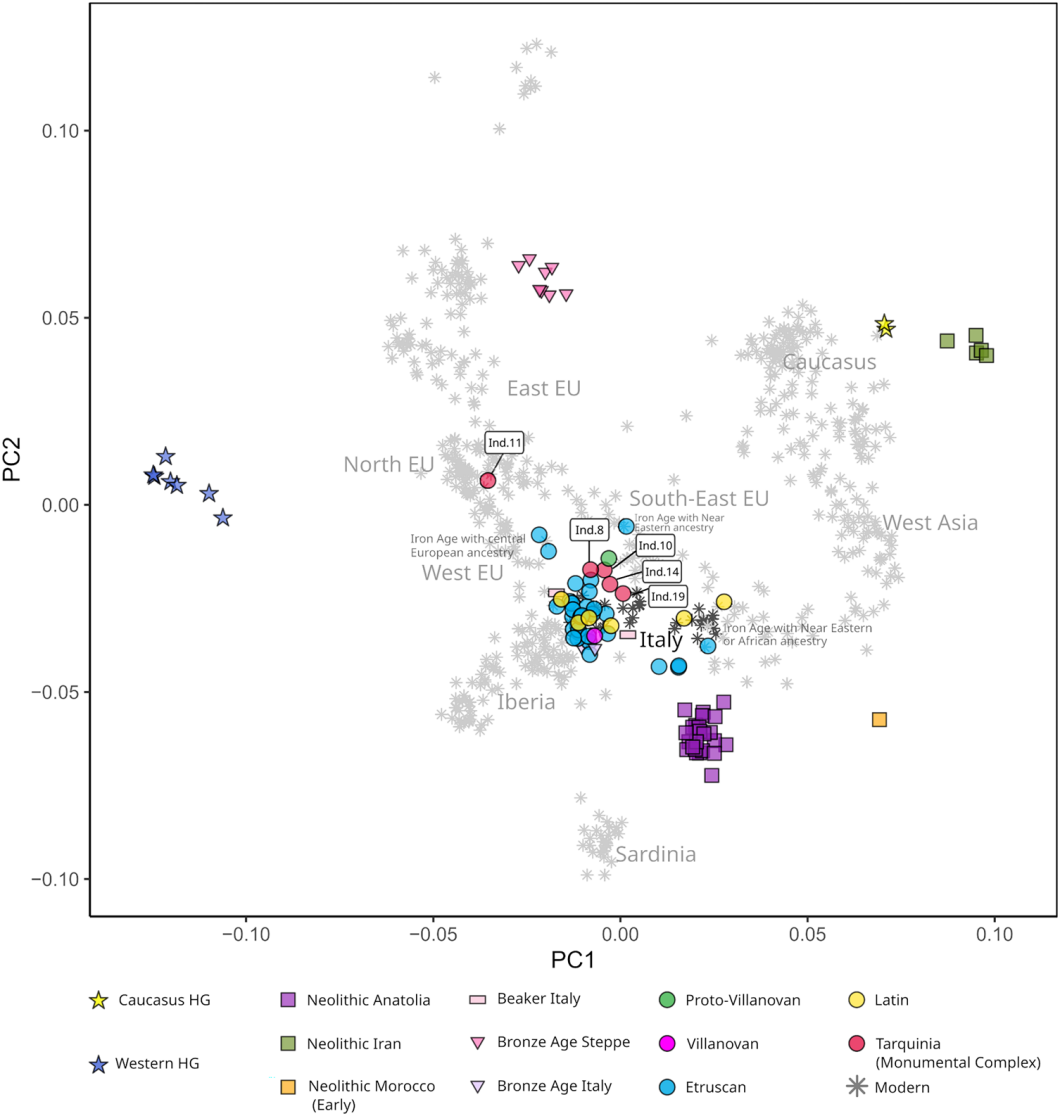
Principal component analysis of ancient individuals^17,18,31–47^ projected onto the genetic variation of modern Europe and West Asia^41^. The modern populations are labelled by broad region. The newly reported individuals are indicated by labels. EU = Europe, HG = Hunter-gatherer.

The majority of Italian Iron Age individuals are projected broadly in the same regions of the PCA (Principal Component Analysis) as modern Italian and west Mediterranean populations. A number of outlier individuals from Etruscan and Latin contexts have already been shown to carry significant amounts of Near Eastern or North African ancestry. Three published individuals from Etruscan contexts (~ 770–200 BC) have a high affinity to modern central European populations^18^. Individual 11 from Tarquinia clusters with populations from Scandinavia, the Baltic Sea, and north Atlantic regions in Europe, with genetic affinities to populations from further north in Europe than has previously been demonstrated in any Iron Age Italian individual. The other Tarquinian individuals cluster with the main group of Iron Age Italian individuals, in the same region of the plot as modern Italy and the central Mediterranean. Their position on one edge of the main cluster of Iron Age Pre-Imperial individuals may be the result of capturing more of the genetic diversity in Iron Age Italian populations (Fig. 4). Details of published ancient individuals used in PCA and admixture modelling analyses can be found in Supplementary Table S10.

#### Admixture modelling

Using the qpAdm program (part of the AdmixTools package^48^) and following from previously published analyses^17,18^, the new individuals with the exceptions of Individual 14 and Individual 11, could be successfully modelled using Italy Beaker as a single source. All, aside from the outlier Individual 11, could be successfully modelled as a two-way mixture of Italy Beaker and YamnayaSamara, which suggests that the Italian Beaker individual used as the source (I1979) may have had insufficient Steppe-related ancestry to serve as a source for Individual 14. The model suggested proportions of 84–92% Italy Beaker and 8–26% additional YamnayaSamara (Steppe-related) ancestry.

The outlier Individual 11 was modelled using European Iron and late Bronze Age populations as single sources. Iron Age populations from Scandinavia and north-west Europe were acceptable single-source fits for Individual 11’s ancestry, confirming her northern European genetic affinity. The accepted models are given in Supplementary Table S5. The results of all tested models can be found in Supplementary Table S11.

#### Diversity of appearance

The pigmentation profiles of the ancient individuals were interrogated using the imputed genotypes and the h-Irisplex-S tool^49–51^. The results are shown in Supplementary Tables S6 and S12.

## Discussion

Tarquinia was one of the largest primate urban sites in Etruria^52^, alongside Vulci to the north and Cerveteri to the south. Unlike nearby cities placed in a volcanic landscape, Tarquinia benefitted from distinctly calcareous, lowland coastal environs which not only supported a distinct agricultural economy, but fortuitously aided the isotope analysis presented here. Tarquinia has long been known from a cultural perspective in terms of its connections and wealth^53^. Bioarchaeology is a multibranch field that integrates various disciplines into the analysis of human remains and their context to reconstruct better their biological, cultural, and environmental experiences in their life course^54^. In this perspective, employing a microhistory approach^13^, this pilot study adds the complementary detail that relates to understanding of some of her population unusually inhumed within the city limits.

Of the six individuals of Tarquinia considered in this study, two were males (Individuals 10 and 19) and four were females (Individuals 8, 11, 12, 14) with age categories ranging from young to old adult. Half of the individuals (8, 10 and 12) presented signs of physiological stress (i.e., enamel hypoplasia, cribriotic lesions, Harris lines) and five (8, 11, 12, 14 and 19) showed signs of mechanical stress (i.e., Schmorl’s nodes, degenerative joint disease, marked entheseal changes). These signs indicate difficult living conditions during growth, from malnutrition to childhood infections, as well as prolonged and strenuous physical activities, potentially related to daily occupations.

All individuals showed traumatic injuries. Half of the individuals presented a traumatic pattern which may be attributed to an accidental event, such as a fall or fractures from occupational activities (Individuals 8, 11 and 12). The other three individuals (10, 14 and 19) exhibited traumatic lesions attributable to interpersonal violence: two individuals (10 and 19) showed lesions that may have resulted from defensive actions, and Individual 14 displayed multiple fractures from at least two temporally distinct traumatic events on areas of the face and neck, typical (although not specific) of assault victims^53,55–58^.

**Figure 5:**
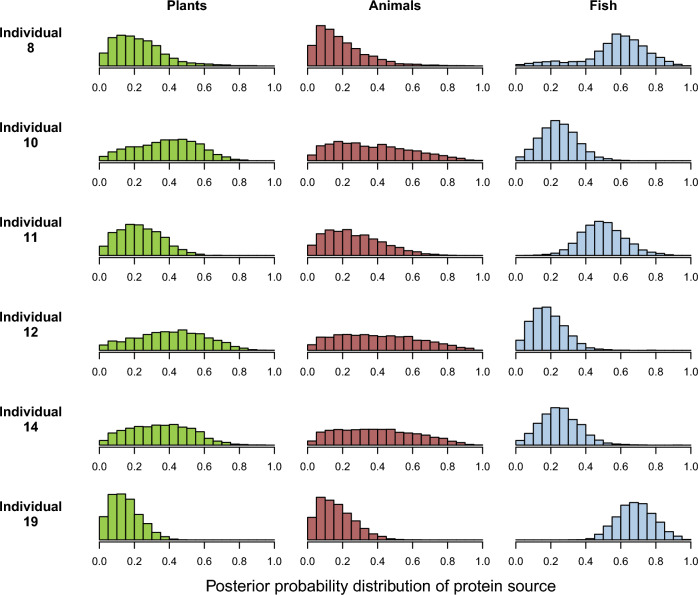
Modelled dietary protein sources based on Nitrogen and Carbon stable isotope measurements from the six individuals compared with flora and fauna from the region.

Palaeodietary reconstruction, through the application of δ^13^C and δ^15^N, has informed on the relative importance of the principal classes of food protein with different chemical pathways. The individuals show a wide range of diet, from a largely terrestrial diet (Individuals 10, 12 and 14) to a strongly marine diet (Individuals 8 and 19). This latter information is also very important for the interpretation of ^87^Sr/^86^Sr isotope analysis, since the consumption of marine food can influence the ^87^Sr/^86^Sr isotope values in human individuals, who show values closer to the higher values of seawater ^87^Sr/^86^Sr values (0.7092 by definition). The Bayesian mixture models of dietary sources suggest marine food was more important as a protein component in the diet of two individuals (8 and 19) than animal or plant foods. These estimates may be improved when more local reference data are collected^59^. Meat consumption, as seen in the later iconography of elite visual culture, may have been a significant part of the diet of Individuals 10, 12 and 14, although significant uncertainties remain (Fig. 5).

**Figure 6:**
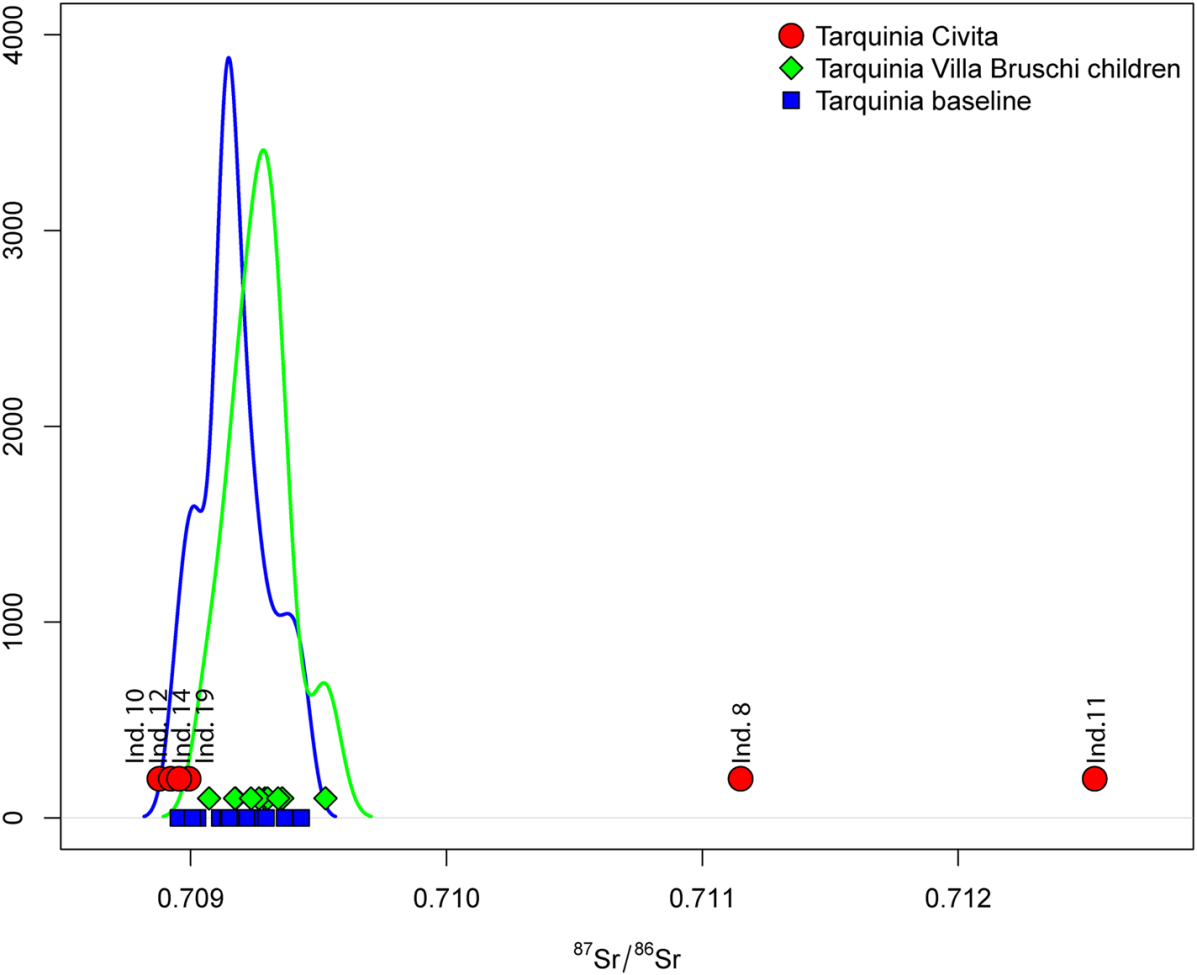
Density plot showing strontium isotope (^87^Sr/^86^Sr) values. Density plot of ^87^Sr/^86^Sr values for the six individuals from Tarquinia Civita, children from Tarquinia Villa Bruschi Falgari and other baseline values (e.g., soil, seed, archaeological fauna). Red dots: Tarquinia Civita individuals; green diamonds: Tarquinia Villa Bruschi Falgari children; blue squares: Tarquinia soil, seed and archaeological fauna values.

Oxygen and strontium isotope values measured from the six individuals buried in the Civita suggest different geospatial histories for these individuals. In the case of the δ^18^O results, the sample number of the Tarquinia Civita is not large enough to assess possible outliers statistically^60^. By comparing the ^87^Sr/^86^Sr results of the six individuals with the BASr values (Fig. 6), it is possible to see that two samples clearly stand out (Individual 8 = 0.71115 and Individual 11 = 0.71253), whereas the others cluster tightly together. Regardless of the small number of samples Individuals 11 and 8 can be considered non-locals, i.e., raised non-locally in the early infancy (Figs. 3 and 6).

Individual 19 (^87^Sr/^86^Sr = 0.70899), who shows significant marine food intake, may have an ^87^Sr/^86^Sr ratio that is dietarily affected and thus may have derived from an area of less radiogenic geology than their value suggests. Indeed, a marine based diet might influence the ^87^Sr/^86^Sr isotope values (see above) (Fig. 5). Individuals 10 (0.70888) and 12 (0.70892) are just outside the local baseline range but only further studies with a larger number of individuals can really clarify their status as outliers. Considering the diverse information that oxygen versus strontium provides regarding the ecological settings where an individual spent their early-life, outlier values for both isotopic markers strengthen the non-local hypothesis, but this cannot be defined with certainty. Therefore we can estimate, with some confidence, that two out of the six individuals from Tarquinia Civita could be considered as raised non-locally (Individuals 11 and 8). It is also possible that some individuals that are consistent with being local could have been raised elsewhere, since the local range is not highly diagnostic and can be attained in various areas of the Italian Peninsula^25^. Indeed, diverse geographical areas could show a similar ^87^Sr/^86^Sr range, making it impossible to distinguish local vs non-local individuals^20^.

With the aid of aDNA sequencing, and by comparison with other studies^17,18^, we can infer that one of these isotopic outliers (Individual 11) not only spent their early life away from Tarquinia as shown by the radiogenic strontium isotopic values, but also had ancestry from a region as distant as the Baltic. The other four that were sequenced in sufficient detail conform convincingly with previous individuals who have been sequenced from first millennium BC central Italy and thus appear to have a more local ancestry.

All the Iron Age individuals (almost entirely from Central Italy) cluster most closely with modern North Italy and Spain, rather than modern central Italy, for reasons outside the scope of this paper but related to population movements post-Iron Age. From the PCA (Fig. 4) we can instead look to their relative position which is with Italy and the west Mediterranean more generally. As in the PCA, each ancient individual is projected independently using underlying modern genetic variation, and since all have a proportion of missing genotypes, their closeness to each other cannot be interpreted further. Rather we could say that all new individuals from Tarquinia (with the exception of the outlier Individual 11) cluster with Italian and West Mediterranean populations, and fall in a similar region of the plot, on the cline of known central Italian Iron Age genetic variation.

Modern perceptions of cultural difference often focus substantially on outward bodily appearance, and it is interesting to note that three out of five of these Tarquinian individuals are predicted to have had blue eyes. So, the diversity of these early populations may have had a potential visual impact beyond any difference in material culture. Such indications need further investigation.

## A synthesis of the archaeological context, bioarchaeological and osteobiographical results in chronological order (Table 1)

**Figure 7:**
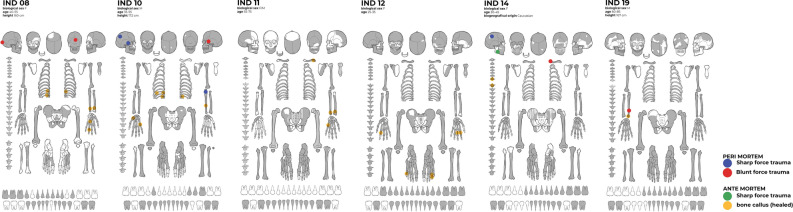
Overview of the state of preservation and location of the injuries of the six skeletons under study (by Lucrezia Rodella).

There is a very precise context for the abnormal inhumations at the ‘monumental complex’. The six skeletons range in date from the ninth to the seventh century BC. They are always connected to ash-rich and fired surfaces, placed in combination with stone blocks or stone structures. All the results are reported in Table 1. A synthesis of the osteological results of each individual is provided in the discussion below and in Fig. 7.

Individual 11 was inhumed in a pit dug into the edge of a hut, a little further east than Individual 10, probably in the first half of the eighth century BC. This chronology depends on that of two fibulae deposited on the skeleton that partially match ^14^C results: 830–790 BC (68%). This burial was covered by ash and stones. The skeleton, with a stature of about c. 163 cm, was that of an old adult with strong evidence of spinal degeneration. Additionally, there was trauma to the left upper limb, suggestive of a fall at some period prior to death. From aDNA analysis, the genetic sex of this individual is female and her genetic affinity is to Northern European, Scandinavian, and Baltic populations. Pigmentation profile analysis indicates she had brown eyes, lighter brown hair, and intermediate skin. The ^87^Sr/^86^Sr isotope analyses (Fig. 6) suggest her early life was in an area different to Tarquinia. δ^15^N and δ^13^C isotope results suggest a mixed fish and terrestrial diet.

Individual 12 was inhumed facing east in a second pit a little further east in the same hut as Individual 11 probably during the first half of the eighth century BC. This chronology relies on that of fibulae deposited on the skeleton and partially matches the ^14^C results: 840–790 BC (68%). Anthropological analyses showed a young adult female and c.163 cm tall. Already at this younger age, there was evidence of spinal degeneration, related to physical activities. This was accompanied by evidence of metabolic stress on the femur and cranium, and some evidence of caries, particularly on the mandible. Healed ante-mortem trauma was evident on the hands and feet. From aDNA analysis, the genetic sex of this individual is female, but insufficient endogenous aDNA survived to do any further analysis. ^87^Sr/^86^Sr isotopic analyses (Fig. 6) suggest her early life might have been spent in Tarquinia or in a region with similar values. δ^15^N and δ^13^C results suggest a substantially terrestrial diet relatively rich in meat (Fig. 5).

Individual 14 was inhumed facing west, and apparently without care, within a square pit next to an elliptical hut. The excavation chronology suggests a protohistoric period, potentially refined by ^14^C to 920–830 BC (68%), but to the ninth/seventh century on stratigraphic grounds. This burial can be linked to the later construction of a sacred building. She was a middle-aged adult female who had evidence of spinal stress and deformation. In addition, trauma was evident on the spine, shoulder area, cranium and mandible, in loci that suggest inter-personal violence. From aDNA analysis, the genetic sex of this individual is female and her genetic affinity is to Iron Age Italian individuals (and Mediterranean populations more generally). Pigmentation profile analysis indicates she had brown eyes, dark brown hair, and intermediate to dark skin. ^87^Sr/^86^Sr analyses (Fig. 6) suggest her early life was spent in Tarquinia or alternatively in an area with similar ^87^Sr/^86^Sr isotope values. δ^15^N and δ^13^C results suggest a terrestrial diet relatively rich in meat.

Individual 19 was inhumed facing east, crouched in a very small, shapeless pit, not far from Individual 14. The excavation chronology suggests the protohistoric period (ninth/seventh century BC), while the ^14^C allows us to limit this date to 740–550 BC (68%), and the ninth/seventh century on stratigraphic grounds. Anthropological analyses showed an old adult male c. 167cm tall and evidence for degenerative joint disease. Enamel hypoplasia was noted on maxillary and mandibular teeth, indicating metabolic stress in youth. There was trauma to the left ulna which could have resulted from an accident or interpersonal violence. From aDNA analysis, the genetic sex of this individual is male, his genetic affinity is to Iron Age Italian individuals (and to Mediterranean populations more generally). Pigmentation profile analysis indicates he had blue eyes, lighter brown hair, and intermediate skin. Y-chromosome analyses suggest he has a J-M241 (J2b2a) Y-chromosome haplogroup. ^87^Sr/^86^Sr isotope analysis (Fig. 6) suggest his early life was in Tarquinia or a region with similar isotope ratios. δ^15^N and δ^13^C isotope results suggest a marine-dominated diet.

Individual 10 was inhumed facing east in a pit dug into the rock at around (750–725 BC). This chronology depends on Geometric pottery fragments deposited close to his skull and broadly matches the ^14^C results: 790–570 BC (68%). This burial had a later cultural marker (a sixth century anchor). Anthropological analyses showed a middle-aged adult man and c. 172 cm tall with marked occlusal dental wear and enamel hypoplasia, which are evidence of substantial chewing and metabolic disturbance during youth. Trauma was detected on the cranium, multiple ribs and right ulna, the latter suggesting attempts to parry blows. From aDNA analysis, the genetic sex of this individual is male and his genetic affinity is to Iron Age Italian individuals (and Mediterranean populations more generally). Pigmentation profile analysis indicates he had blue eyes, dark brown hair, and intermediate skin. His Y-chromosome haplogroup is J-M12 (J2b). ^87^Sr/^86^Sr analyses (Fig. 6) suggest his early life was probably in Tarquinia or in a region with similar isotopic values. δ^15^N and δ^13^C istope results suggest a relatively low marine diet.

Individual 8 was inhumed in a large pit excavated in the bedrock around 650 BC; this traditional chronology is confirmed by ^14^C: 660–400 BC (68%). In this pit, one more individual (7) was superimposed on this skeleton, which was chosen for this stratigraphic relationship and a series of cultural markers. Anthropological analyses showed a middle-aged adult woman c.160 cm tall with frequent use of the limbs for occupational practices as evidenced from the marked entheseal changes of the hand phalanges. Likely evidence of consistent chewing of hard foods was observed in the form of osteoarthrosis of the temporomandibular joints and high severity of tooth wear. Dental abscesses and enamel hypoplasia indicated an event of metabolic disturbance during youth^61^. Several traumatic lesions were found on the ribs, left arm and cranium. From aDNA analysis the genetic sex of this individual is female, her genetic affinity is to Iron Age Italian individuals (and to Mediterranean populations more generally). Pigmentation profile analysis indicates she had blue eyes, light brown hair and pale-intermediate skin. ^87^Sr/^86^Sr isotope analyses (Fig. 6) suggest her early life was in a region different to Tarquinia. δ^15^N and δ^13^C results suggest a marine dominated diet.

Further details of the analyses are reported in the Supplementary Notes.

## Conclusions

The six skeletons analysed here were already marked as different by their funerary rite and their place of deposition. The study of the osteological biographies of some of the unusual individuals, combined with their biochemical signature, has highlighted their difference on a range of criteria. The individuals from the urban centre were inhumed, had distinctive medical histories, often suffered violent deaths, were diverse amongst themselves in appearance, were likely to have a history of mobility that was, in at least one case, apparently quite distant and were memorialised by ideological markers placed above them. More generally, these individuals give insights into the level of violence and poor living conditions that were inherent in the social and political changes at the foundations of the early city. The evidence suggests that the six individuals had varied diets dominated by cereals and marine fish, where the consumption of meat and plants varied substantially, showing no gender preference and complementing impressions seen in the later visual culture of these communities.

In the eloquent spirit of Pirandello, from which the title has been taken, the six individuals embedded in the stratigraphy of the Civita plateau have thus unwittingly offered, through osteological and other scientific analyses, a narrative of themselves and their living conditions. The exceptionality of the type of burial and its memorability in a sacred area help to qualify them as individuals selected by the community for rituals aimed at consolidating the community around the ancestral core of the ‘monumental complex’ whose importance had already been grasped by the archaeological research carried out over the last forty years.

In the course of further research, we will be able to discover if they share these characteristics with the other fourteen inhumed skeletons at the monumental complex and to what extent they differ from the vast majority of the community. We will also be able to examine the characteristics of those buried in standard cemeteries, subject to the limits imposed by cremation.

## Methods

The ambitious multi-facetted methodology employed in this study necessitated the application of macroscopic and molecular methods in a range of labs across Europe. The multi-scalar datasets were coordinated and integrated by the project leads. In instances, where analyses were undertaken at different labs (e.g., ^87^Sr/^86^Sr) data should be directly comparable because of the use of international and in-house calibrated standards (see Supplementary Notes for more detail).

### Osteoarchaeology

Anthropological analysis included the estimations of sex, age-at-death, population affinity, stature, as well as pathological and traumatic study of the six individuals. These data are summarised in Table 1, Fig. 7 and in the Supplementary Notes.

### Dating

Chronological definition is important for the current project since our aim has been to study a contemporary community at the moment of its early formation in the tenth to ninth century BC and its ongoing development. For this we have combined traditional archaeological typo-chronologies with accelerator mass spectrometry (AMS) radiocarbon dating, calibrating using the most recent data and Bayesian statistics employing contextual knowledge of the excavators and an assessment of associated material culture. The analysis of six skeletons was performed at the 14CHRONO Centre, Queen’s University Belfast using described protocols^62^. The analysis of further four skeletons was undertaken at the University of Lecce. For full details see the Supplementary Code, Figures and Tables supplied in the Supplementary Notes.

### Carbon (δ^13^C) and nitrogen (δ^15^N) isotope analyses for palaeodietary reconstruction

Carbon and nitrogen isotopic analysis of bone collage (tooth roots; Supplementary Table S2) was undertaken at the same time as the dating. For full details of the methodology see the Supplementary Notes. The analysis was performed at the 14CHRONO Centre, Queen’s University Belfast, using isotope ratio mass spectrometry (IRMS)^62^. Mixture modelling was done with *simmr*^63^ using trophic enrichment factors of 0.8 ± 0.5 ‰ for ^13^C and 4 ± 1 ‰ for ^15^N and the sources baseline for the central Mediterranean^59^.

### δ^18^O isotope analysis

Mass spectrometry was performed in the Leibniz Laboratory at the University of Kiel (Germany). Enamel powders were reacted with H3PO4 at 75C under vacuum on a Kiel IV carbonate preparation device interfaced with a Finnigan MAT 253 mass spectrometer. Samples were referenced against two international carbonate standards, NBS-19 (δ^13^C =  + 1.95‰ V-PDB; δ^18^O =  − 2.20) and IAEA-603 (δ^13^C, + 2.46 V-PDB; δ^18^O =  − 2.37 V-PDB) and two internal enamel standards (CM1 and ER 1).

For further details of the extractive methodology used see the Supplementary Notes.

### ^87^Sr/^86^Sr isotope analysis

As for inhumations, tooth enamel was sampled using established protocols^64^ and described in further detail in the Supplementary Notes.

The assessment of the local ^87^Sr/^86^Sr baseline was based on a multifactorial approach^20,64^ taking into account: (a) the geological and isotopic composition of the area around Tarquinia, (b) the assessment of the local baseline measuring diverse modern and archaeological samples from around the site, (c) the assessment of the ^87^Sr/^86^Sr range for the youngest individuals from the contemporary Iron Age necropolis of Tarquinia Villa Bruschi Falgari^65,66^, namely cremated young children (YC) and older children (OC) aging from 1 to 10 years of age. Petrous bone and tooth enamel in subadults form shortly before death, and, consequently, a limited possibility of residential mobility can be inferred^67^. As for (b), 16 samples which included faunal archaeological specimens from the Civita (n = 6), modern seeds (n = 5), soil samples (n = 4) and a palaeo-soil (n = 1) were collected and analysed (see Supplementary Table S3). As for (c), ten cremated human petrous bones and tooth enamel were selected from the necropolis of Villa Bruschi Falgari, which will be part of a broader study that will be published elsewhere. The petrous bone was sampled following the VUB (Vrije Universiteit Brussel) protocol^68^ to ensure precise sampling of the otic capsule, which does not remodel after development, thus providing a temporally resolved early life signal. The method is described in further detail in the Supplementary Note. Baseline and tooth enamel samples were analysed at the Frankfurt Isotope and Element Research Center (FIERCE)^64,69^. Cremated tooth enamel and petrous bones from Tarquinia Villa Bruschi were analysed at Cardiff Earth Laboratory for Trace element and Isotope Chemistry (CELTIC).

### Oxygen (δ^18^O) and strontium (^87^Sr/^86^Sr) isotope analysis for individual mobility

Strontium and oxygen isotope analyses are used to detect human mobility in the past^60,70–72^. Human tooth enamel from six individuals from Civita were selected for δ^18^O and ^87^Sr/^86^Sr analysis (Supplementary Table S3). Ingestion of breastmilk influences oxygen isotope values^73^, so we selected second permanent molars (M2; n = 5) for analysis. The M2 was not available, however, for one individual (Individual 8; M1). Enamel formation in the second molar begins around three-four and is completed by eight years of age^74^. The first lower M1 crowns usually form before birth, as witnessed by the presence of the neonatal line (i.e., an incremental line forming at birth^75^) until three years of age.

### DNA extraction

Detailed descriptions of DNA extraction, sequencing, sex determination, mitochondrial analysis, Y-chromosome analysis, contamination estimation, principal component analysis, qpAdm admixture modelling, genotype imputation, pigmentation profile analysis and kinship analysis can be found in Supplemental Information.

### Ethics approval

The current research was accomplished following the relevant regulations for the treatment of ancient human remains. Permits for osteological and isotopic analyses were granted by the Soprintendenza Archeologia, Belle Arti e Paesaggio per la Provincia di Viterbo e Per L'Etruria Meridionale.

### Supplementary Information

Supplementary Information 1.

Supplementary Figures.

Supplementary Tables.

## Data Availability

Raw FASTQ and aligned BAM files are available through the European Nucleotide Archive under accession number PRJEB74104. All other data are fully available within the Supplementary Information. Other data are fully available within the Supplementary Information.
