# Supplementary material for: Bioarchaeology aids the cultural understanding of six characters in search of their agency (Tarquinia, ninth–seventh century BC, central Italy)
Source: Sci Rep. 2024 May 28;14:11895. doi: 10.1038/s41598-024-61052-z (PMC11133411; doi:10.1038/s41598-024-61052-z)
Supplement: Supplementary file 1 — Supplementary Information 1. [file 41598_2024_61052_MOESM1_ESM.docx]

**Bioarchaeology aids the cultural understanding of Six Characters in Search of their Agency (Tarquinia, ninth – seventh century BC, central Italy)**

Bagnasco, G.^1^*, Marzullo, M.^1^, Cattaneo, C.^2^, Biehler-Gomez, L.^2^, Mazzarelli, D.^2^, Ricciardi, V.^2^, Müller, W. ^3, 4^, Coppa, A.^5^, McLaughlin, R.^6^, Motta, L.^7^, Prato, O.^8^, Schmidt, F.^9^, Gaveriaux, F.^10^, Marras, G.B.^9^, Millet, M.A.^11^, Madgwick, R.^12^, Ballantyne, R.^13,14^, Makarewicz, C.^15^, Trentacoste, A.^15^, Reimer, P.^16^, Mattiangeli, V.^17^, Bradley, D.G.^17^, Malone, C.^16^, Esposito, C.^18^, Breslin, E.M.^17^, and Stoddart, S.^9^*

*corresponding authors

email: GB - giovanna.bagnasco@unimi.it

SS - [ss16@cam.ac.uk](mailto:ss16@cam.ac.uk)

| ^1^ | Dipartimento di Beni Culturali e Ambientali, CRC “Progetto Tarquinia”, Università degli Studi di Milano, Milan, Italy. |
| --- | --- |
| ^2^ | LABANOF (Laboratorio di Antropologia e Odontologia Forense), Università degli Studi di Milano, Milan, Italy. |
| ^3^ | Institute of Geosciences, Goethe University Frankfurt, Frankfurt am Main, Germany |
| ^4^ | Frankfurt Isotope and Element Research Center (FIERCE), Goethe University Frankfurt, Frankfurt am Main, Germany |
| ^5^ | Dipartimento di Storia Antropologia Religioni Arte Spettacolo, Sapienza Università di Roma, Rome, Italy |
| ^6^ | Hamilton Institute, Maynooth University, Maynooth, Ireland |
| ^7^ | Department of Classical Studies and Program in the Environment, University of Michigan, Ann Arbor, Michigan, USA. |
| ^8^ | Institute of Archaeology, UCL University College London, London. |
| ^9^ | Magdalene College, Cambridge, UK. |
| ^10^ | Kelsey Museum of Archaeology, University of Michigan, Ann Arbor, Michigan, USA. |
| ^11^ | School of Earth and Environmental Sciences, Cardiff University, Cardiff CF10 3AT, Wales, UK |
| ^12^ | Cardiff School of History, Archaeology and Religion, Cardiff University, Cardiff, Wales, UK. |
| ^13^  ^14^ | School of Archaeology, University of Oxford, Oxford, UK.  Department of Archaeology, University of Cambridge, Cambridge, UK |
| ^15^ | Institut für Ur- und Frühgeschichte, Christian-Albrechts-Universität zu Kiel, Kiel, Germany. |
| ^16^ | School of Natural and Built Environment, Queen’s University Belfast, Belfast BT7 1NN, UK. |
| ^17^ | Smurfit Institute of Genetics, Trinity College Dublin, Dublin2, Ireland |
| ^18^ | Dipartimento di Beni Culturali, Alma Mater Studiorum, Università di Bologna, Ravenna, Italy |

# **Supplementary note**

**The historical and archaeological background**

The Etruscans were one of the great European Civilisations of the first millennium BC.^1,2^ Their cities principally occupied the central Italian region bounded by the Tyrrhenian sea to the west, the River Arno to the north and the River Tiber to the south and the east, with some extensions north into the Po Valley, east into the Apennines and south into Campania. Tarquinia was one of the primate cities on the coast, located between Vulci to the north and Cerveteri to the south. Considerable research has been undertaken on the cultural development of both the Etruscans in general and on the city of Tarquinia in particular^2,3,4,5,6^. Most notably, Tarquinia is known for painted tombs^7^ and ritual places^8^, and the skeletons studied here are drawn from one of these ritual places. The rural settlement of a number of cities including parts of the territory of Tarquinia have also been studied^9,10,11^, creating a balance between knowledge of the elite and the sustaining population. Studies of the life sciences of these urban communities have seldom been undertaken, outside Tarquinia^12, 13, 14^. This pilot project has now brought together updated methodologies and studies of botanical, animal, and human remains, combined with studies of geoarchaeology, isotopes and ancient DNA (aDNA). These studies promise to provide new understandings of a civilisation that has been relatively ignored in comparison with their near contemporaries, the Latins and Greeks.

**The methodological background**

This work presents several analyses conducted in different laboratories and according to different methodologies. After a 40-year archaeological research carried out by archaeologists together with experts of other disciplines within the University of Milan, especially in the field of anthropology, a new phase of research was established through the joint undertaking of Science@Tarquinia carried out by the Universities of Milano and Cambridge. This project attracted a number of different disciplines, especially in the field of isotopes and aDNA. The authors have amalgamated all this information with the precise and powerful aim of reconstructing the lost profile of Tarquinia from many categories of evidence without the constraints imposed by information from classical written sources.

The constraints on this large project are financial and the time and energy devoted towards the coordination of the different schedules and resources of every single researcher.

**The geological and pedological context of the Tarquinia Civita**

The area of Tarquinia is dominated by Pliocene marine sedimentary rocks^15^. Calcarenite and limestones, locally called “Macco”, constitute the plateau-like relief of the Tarquinia Civita. The limestone unit is intercalated with a deposit of sands and clayey sands, underlain by Early Pliocene mudstones. According to the recently established Sr isoscape map of Italy^16^, the local bioavailable ^87^Sr/^86^Sr range for this area is between 0.7091 and 0.7094 (Supplementary Figure S1), within 5 km radius.

The Tarquinia ‘monumental complex’ rests on a plateau-like calcareous hill, surrounded by steep slopes on all sides except the east (Figure S2). The lithologies of this territory originate from the Pliocene marine transgression that occurred between 5 and 3 million years ago, after the latest Miocene Messinian stage ^17,18,19^. Thick mud units were deposited in epibathyal environments within these basins, represented by the 'Argille Azzurre Formation' exposed at the base of the Tarquinia Civita. These muddy sediments are overlain by calcareous and sandy sediments. The 'Macco Unit', exposed on top of Civita Hill, consists of calcarenites abundant in marine fauna like bivalves and echinoids. It alternates with competent limestone and sandy mudstone units showing horizontal bedding structures, overlying the 'Argille Azzurre Formation'. Above the 'Macco' lies the 'Pian della Regina Unit', consisting of sands, clayey sands, and mollusca-rich sands. The clayey units, in particular, show significant oxidation^17^.

The erosion of the less resistant 'Pian della Regina Unit' in the areas of the archaeological site exposed the top of the calcarenite 'Macco Unit', creating the plateau-like Civita Hill^19^. The regional soil map^20^ indicates that the predominant modern soil type covering Tarquinia's hills is a Calcaric Cambisol. This soil is characterized by incipient soil formation, weak transformation of the parent material into soil horizons, and intense precipitation of secondary carbonates, attributed to the xeric-dry climate. In addition, a truncated palaeosol with a Bt-2Btk profile was identified in sector Q of Tarquinia Civita. This palaeosol was extensively eroded in the southern part of the site, where archaeological structures and deposits lay directly on the ‘Macco Unit’, so that it is preserved for a maximum thickness of 60 cm in the north-western area. In this palaeosol, Etruscans excavated a number of pits of different dimension which represent the current structures in use in the sacred area, but this relationship between a burial pit and a palaeosol has not yet been proven elsewhere. The B-horizons show a well-developed angular blocky structure with a sandy clay texture, with colours ranging from reddish brown (5YR 4/4) to red (2.5YR 4/8), with a marked increase in pedogenic carbonates downwards. Under microscopic analysis, the Bt-horizon exhibits a clay loam texture with a coarse mineral component of moderately sorted rounded fine sands composed mainly of mollusc fossils, calcarenite and mudstone fragments, quartz, and traces of sanidine, plagioclase, and trachytic rock fragments. Pyroclastic materials are likely to have originated from alluvial deposition by fluvial channels on top of the ‘Macco Unit’, if not by primary volcanic activity. Alluvial deposits are not mapped on the area of the Civita, but they do outcrop on the opposite side of the River Marta Valley at the same height and longitude as the Civita plateau (165 m). More precisely, coarse to fine sediments of fluvial to lacustrine facies rich in volcaniclastic deposits are mapped in the Tarquinia area as belonging to the ‘Roccarespampani Unit’, dated to the Middle Pleistocene^15^. Based on macroscopic and microscopic features, this palaeosol profile is classified as a truncated re-calcified Terra Rossa-like soil (Calcaric Luvisol), resulting from diverse pedogenesis cycles under varying climate and environmental conditions. Together with the presence of igneous minerals (e.g. sanidine and trachytic fragments), this pedosedimentary sequence suggests a rather complex soil formation history that needs further analysis.

**The Archaeological sequence of the ‘monumental complex’^21^**

From the end of the Protovillanovan until the end of the Villanovan period (corresponding to the Iron Age: from the late tenth to the eighth century BC), the site was intensively occupied over the entire excavated area of about a quarter of a hectare. From the ninth century BC onwards, a natural cavity became the central focus: layers of ashes and a series of small pits, containing ceramic sherds and animal bones. The deposition of a distinctive child, in a quadrangular area close to the cavity (*area alpha*) was part of this emerging process. The osteoarchaeological analyses revealed that the child (not one of our six skeletons studied here) was about eight years old, seemingly encephalopathic and epileptic^12^. It was buried on a thin layer of clay-like soil and covered with a similar layer, with few grave goods, although it was found with fragments of deer antler, some of them partially worked, and two bronze objects placed on its chest as personal belongings (a pendant and part of a bronze pin)^12^. Area alpha was also distinctive for the quantity of red deer antler fragments (see below).

In the second half of the eighth century BC, the natural cavity was surrounded by structures of perishable material cut into the ground surface. The study of the inhumed skeletons is now in progress, and the six characters presented here form part of that study (Fig. 2).

Various *Macco* (compressed calcareous material) layers of circular or subcircular shape composed of layers of earth, clay and charcoal were deposited on top of these burials as markers (*monumenta*).

At the end of the eighth century BC, walls in blocks of local *Macco* were built over the preceding structures of perishable material cut into the ground surface, surrounding the natural cavity. During the following Orientalizing period (from the first quarter of the seventh to the first quarter of the sixth century BC), Mediterranean contacts can be detected in the technical features of masonry (pilaster-wall linked by sections of smaller stones) of the temple (*edificio beta*) erected to the east of the natural cavity. The very first plan of the *edificio beta* was also influenced by Eastern models: it was precisely orientated, (measuring 6.5 x 11.0 m in size) and was divided into two axial rooms, the inner containing a bench/altar in one corner, delimited on one side by large stone blocks and by a canal carved in the soil to convey liquid to the natural cavity. A set of three bronze objects, materialised symbols of power, were placed folded one above the other (the famous “bronzes of Tarquinia” (axe, *lituus*, shield)) in front of the main entrance to the east, above a pre-existing ovoid hut. These were located in a large pit, alongside another similar pit, that was less deep, with animal bones and vessels in *impasto* (hand-made) ware and early *bucchero* (a burnished, reduced, and thus shiny black, fine pottery). After the mid- seventh century BC, the *edificio beta* was flanked by two adjacent courtyards and surrounded by an imposing precinct formed by pilaster walls; of a design that employed the golden section to define the relationship between the short and long sides. This plan mirrored the principles of the most important buildings of first the Near East, and then of classical Greece^22^.

Between the end of the seventh and the beginning of the sixth century BC, the final phase of the Orientalizing period was registered in the ceramic assemblage. The basic characteristics of this part of the city were established in its first crucial phases of foundation, leading to a permanent structuring of the ‘monumental complex’ in the archaic period, during the first half of the sixth century BC. The spaces inside and outside the precinct were monumentalized, refurbishing and reinforcing the previous walls and floors and adding new arrangements to improve the general setting of the area.

In the late archaic period (early fifth century BC), the monumental complex was redesigned and the area between the *edificio beta* and *area alpha* was dismantled and substituted with a North-South pathway, supplied with an underground tunnel (*cuniculus*) covered with stone slabs over the natural cavity. A new cavity was introduced into use in the north-east area of the ‘monumental complex’ where a well directly dug in the bedrock was monumentalised by a blind arch.

Recent discoveries have enhanced our knowledge of the Hellenistic period, already indicated by a mosaic floor. The upper part of the well surmounted by the arch was sealed by fragments of a skilled terracotta high relief plaque with a warrior in battle dated between within the third century BC. It was probably part of the decoration of a small building in use at the ‘monumental complex’ with space for rituals performed by a substantial number of persons. Large fragments of black glaze and *impasto* pottery, *ollae* and lids, datable to a similar span of time, were deposited and sealed within the well in the mid- second century BC. Most of the pottery was marked with *sigla* (short inscriptions) bearing a cross inscribed in a circle and a complete or abbreviated single name in Etruscan and Greek and inscribed by different hands.

The study of the later phases of the monumental complex is in progress and not the central focus of this article. An impressive hypogeum formed by a circular chamber with six pillars supporting its ceiling with an entrance staircase was built in the same area probably during Augustan (early Imperial Roman) times. During the eighth century AD (dated by radiocarbon), it was used for the deposition of more than thirty skeletons which are also not part of the current study.

**The detailed context of each** **skeleton**

**Individual 8**

Votive pits with offerings were dug in this area (alpha) from its very earliest development. An encephalopathic child was buried here^12^, followed in turn by three other neonatals, and then reshaped by a roughly N- S wall, that rested on earlier stones, with the function of highlighting what was buried below. Likewise, a twofold rite was performed with the multiple scope of underpinning the building of the quadrangular structure, forming the new alpha area and of obliterating what laid below. Two vases were set between the foundation stones roughly in the centre of the N-S wall and above the layers corresponding to the previous phases of activity. They contained large quantities of cereals (barley, emmer and wheat), legumes, fruits and some weeds, alongside fish and suckling pigs. This comprised a skeleton of an adult woman (Individual 7) under an extensive layer of burnt *Macco* that showed extensive burn marks, archaeologically dated to the Middle Orientalising period (c. 650 BC). The body lay supine exactly below the N-S wall with the head to the east in a pit without grave goods; skull supported by a quadrangular shaped stone. Contemporary layers contained a gem engraved with a character evoking Herakles and the Nemean Lion^23^ and three large fragments of Geometric pottery, showing the extensive cultural links of the period. Individual 8 was in a deeper pit below Individual 7, facing east and may have been propped up on an item of perishable material. Arms, placed along the hips and hands, were resting on the body. There were no grave goods, except for a large and almost intact shell (Pecten) which probably was originally above the eye. Previous osteoarchaeological analysis defined this body as that of a man. The dating of the two depositions, which took place at the same time, can be traced back to the Middle Orientalizing period (within the first half of the seventh century BC).

**Individual 10**

This body was found in a part of the site north of the sacred cavity, in the F sector. It was below the archaic phase of floor 535 which rested on the wall 14a, covering its foundations. In this context, a fragment of an anchor had been placed near the wall. It was inscribed with a Cypro-syllabic short text probably connected to the function of the anchor^24^.

This element probably served as a marker for the deposition buried below. On the other hand, it recalls the direct connection with the sea and, because of the shape and inscription, the close contact with the Aegean-Anatolian world. This piece of stone anchor probably helped memorialize the location placed over a series of previous floors, which dated back to the eighth century BC.

A mass of clay mixed with *Macco* covered a pit in which individual 10, an adult male, had been placed supine with the head to the northeast. The skull was reclined on the right side and rested on stones arranged as support. The right arm rested on the body while the left arm was at the side. The legs were stretched out. Fragments of a Geometric vase from the half of the eighth century BC were placed near the skull and on the chest. And, as in the previous case of Individual 8, a shell was found in the small pit near the individual's legs.

**Individuals 11 and 12**

These two inhumations were found a little further east in the O sector. They were earlier in date, since, at the transition between the ninth and eighth century BC, a large elliptical structure was dug into the rock. It is probably the lower part of a large hut, which measures about 8 metres by 4 at its greatest extension. Two pits were dug in its southern part. The first deposition (Individual 11) was located at the western edge of the hut. The skeleton is not well preserved and appears to have been damaged by the activities that subsequently took place in the area.

The direction of the body was always E-W, with the head pointed towards the E. Arms were stretched out to the sides and the out-stretched legs were cut by a disturbance. The body was buried with a bronze needle and an arc fibula on the ribs dating to earlier than 750 BC.

An adult woman, slightly younger than the previous examples, was buried inside a second pit set a little further east in the bottom of the hut lying E-W. The head was pointed to the E and the face turned towards the North. As in the previous depositions, the right arm rested over the body, while the left arm was extended; the legs were extended and the feet rested on the edge of the pit. Some bronze fragments, including two bronze fibulae, were found close to the body. One of these is of Campanian type that can be dated earlier than the middle of the eighth century BC.

Subsequently, the two pits were covered with thin layers of ash, which were the first filling of the hut. The eastern body was also covered with clay and *Macco* and shortly afterwards the entire structure was filled with very burned soil. An intact vase was positioned between the two depositions in the south-western part of this layer. Shortly afterwards, a small offering in memorialisation of the burial below was placed in a small pit, containing only a few stones, fine ash and larger fragments of charcoal.

Probably during the same phase, the entire structure was filled with another scattered and thick layer of ash and charcoal overlain by a thick layer of *concotto* (baked clay). This totally sealed the structure which was then completely abandoned. During the Orientalizing period, two huge votive circular pits were dug in the western part and in the centre of the hut. Another elliptical structure was placed close to the eastern edge of the hut. All these structures destroyed a part of the previous Villanovan hut and more particularly the western pit destroyed the individual 11’s feet. During the archaic phase, a rectangular structure built with stone walls and a *Macco* floor was placed over a layer of ash in the centre to provide activities of memorialisation of the locale.

**Individual 14**

Moving to the western area of the excavation, the last two individuals were found a short distance from one other, within a dense Villanovan phase of activity. Individual 14 was placed within a square pit dug for this purpose. The head pointed West, with the right arm rested on the outer edge of the pit, and the left hand placed under her head. The body was turned to its right side, with the legs partially extended, albeit in a relaxed position.

The burial appears framed within a dense network of post holes and Villanovan walls. In particular, its close relation with an elliptical structure, which could be interpreted as a hut, is very significant. In fact, the cut of the structure touched the edges of the pit of Individual 14 and the fill rested gently on the right arm, respecting it. This structure is still to be investigated, because a temple with stone masonry and a spectacular decorated architectural terracotta roof was built on it in the first archaic period.

In this case too, the close vertical spatial relationship that links the structures of different chronological phases, appears evident and suggests that the sacred role of the hut has been transferred to the archaic temple.

Consequently, the burial of the Individual 14 represents another case of memorialisation.

**Individual 19**

This adult man was placed inside a very small and shapeless pit. The body was crouched and folded in a foetal position turned onto the right side. The back was compressed, and the legs were bent and pressed as if they had been forcefully squeezed against the body. The arms were folded over the chest and the joined hands were in front of the face. The impression is that the body was held in this position by materials that have since decomposed. As in the previous case, the pit was filled with what appeared to be its own back-fill. The burial was indicated on the surface by a layer of *concotto* and Macco, while remains of kilns were found below. Individual 19 was surrounded by the same dense network of post holes and Villanovan walls as Individual 14. The body was also found near an elliptical structure carved into the rock, which can be interpreted as a hut.

The chronology of the context is unclear since the burial lacked grave goods whereas the hut was from the first decades of the eighth century BC. The overlying layers could be as late as the archaic age and because of the Hallstatt radiocarbon plateau, radiometric dating on the individual did not clarify the situation. Further study is in progress. What is clear, in any case, is the close relation between huts, post-holes and ditches, temple, sacred area and the burials.

**Overall considerations**

The abnormal inhumations at the ‘monumental complex’ played a leading role in the design of the sacred space and followed one another from the Final Bronze Age up until at least the archaic age according to very precise practices and design. They are always connected to ash and *concotto* altars and were placed in combination with stone blocks or stone structures of considerable ritual value.

From this point of view, some constant features are particularly evident. They allow us to shed specific light on the religious beliefs of the local culture. First of all, there is continuity with actions and practices that have taken place over time. The ritual sealing of the pits, the stone markers, the vases above depositions, and the number of elements used to highlight the buried evidence over time are clear example of this.

These activities initially consisted of specific repeated actions that were always the same and in the same points in space: small votive pits dug into the ground where fires were lit and plants and animals offered. These include a number of worked and unworked deer antlers. The pits were then sealed by layers of ash, crushed and burned *Macco*. They remained visible on the surface for several decades and contributed to the consistent pattern of memorialisation.

The repetition of these elements over the course of time made it possible to identify an early cult of the female divinity venerated at the 'complex' with remarkable clarity. Solid clues for her attributes were detected even at the beginning of the sequence when her name Uni was made clear^25^. Indeed, after numerous studies dedicated to these issues, it is now possible to establish some key points in our cultural interpretation. First of all, they concern the high probability that pits and structures of ashes and *concotto* were linked to the cult of the most remote ‘unnamed goddess', good and favourable, protector of the underworld, of hunting, of nature, of the lunar cycles and the animals ^26,27^. Veneration through offerings, libations and multifaceted practices can be followed through the composition and distribution of these structures over the course of time. These practices remained unchanged from the Bronze Age up until the archaic phase, whilst becoming more and more structured over the decades. They can therefore be considered real *en plein air* altars, made up of various materials and features that remained unchanged from generation to generation. They acquired a more defined appearance over time, thanks to their increased frequency^28^.

For this reason, all the depositions of the 'monumental complex' were part of the rituals of the divinity, precisely because of their special treatment in the sacred area. They find a specific place in the evolution of the primitive cult dedicated to the female divinity of the 'complex'. This cult was transmitted over the centuries from the numinous past of the Bronze Age, then to characterize the ritual practices of the more evolved phases with greater and greater information.

**Broader osteological comparisons**

It is interesting to note that based on palaeopathological analyses using similar methods, only 40% of Roman Milanese women showed signs of mechanical stress^29^. Although Roman Milan is both geographically and temporally distinct from Etruscan Tarquinia, this result, dissimilar from other sites in Antiquity, may indicate that the females of Tarquinia were very active and more physically involved in everyday life than what was observed for their Roman counterpart. For instance, only 34% of the skeletons from Roman Milan showed signs of trauma^29^. Similarly, about 35% of individuals from a mass grave found in ancient Ephesus (Turkey) linked to gladiator tombstones showed traumatic lesions. It is important to note that Roman gladiators, depending on the type, wore protective gear providing protection of the head, upper limbs and dominant leg, in addition to the shield^30^. Other sites in Roman border provinces (Britannia, Dalmatia and Pannonia) show frequencies of trauma occurrence varying between 8% and 13%^31^. These results show that visible trauma rates in Tarquinia were exceptionally high and may be related to specific occupational activity or violence in Etruscan Tarquinia.

**Osteological methods**

Sex estimation relied upon dimorphic morphological traits of the pelvis and cranium ^32, 33, 34,35^ and metric analysis ^36,37^. Age-at-death estimation was based on degenerative changes at the pubic symphysis ^38^ auricular surface^39,40^ and acetabulum^41^, sternal end of the fourth rib^42^ and radiological measurements of the dental pulp^43^. Population affinity was estimated from morphometric traits of the cranium^44, 45^. Pathological and traumatic analysis was performed following palaeopathological standards^46, 47, 48, 49^ and forensic methodologies ^50, 51, 52^. Tooth numbering system followed the ISO 3950 notation. The skeletons underwent conventional radiographic imaging using a Poskom PXM-40BT and an X-DR L WiFi with the following technical parameters: 50 kV and 4 mAs and then acquired using Examion® software.

**Agriculture**

We outline here some of the details of the recovery of plant and animal remains from the same deposits. A great investment has been made in flotation of sediments from the archaeological deposits, making this site more important than even pre-Republican Rome, for the statistical validity of the recovery of plant remains. As an illustration of this, more than 30,000 carbonised and mineralised plant macrofossils have been recovered from secure stratigraphic levels, by systematic sampling and flotation of more than 1000 litres of sediment employing a machine assisted flotation system that captures both the light and

heavy fraction. Animal bones are more commonly recovered from Etruscan sites, but also here provide a reasonable sample. All these data can then be compared with the isotopic results. It is noteworthy that the isotopic results give a more reliable understanding of the ingestion of food, bringing plant and marine resources into greater prominence. It is also to be noted that foxtail millet which might affect the isotopic results is of relatively low frequency, albeit present.

**Plant remains**

Archaeobotanical sampling at the ‘monumental complex’ has so far produced a rich and well-sequenced assemblage of carbonized food-plant remains, which allows assessment of certain features of diet during the periods contemporary to the burials, which can be set within the broader context of Etruscan plant consumption^53^. We have, whenever possible, applied a systematic 20 litre sampling regime to all deposits, to provide a consistent approach to recovery, aiming to minimize bias within the limits of preservation which is primarily by carbonization and secondarily by mineralization.

Cereal grains are most abundant at all times. Amongst these, emmer and barley are especially prominent, as they together account for at least 60% of the grains during any occupational phase, but possibly even up to 80-90% when the majority of unidentified wheats are accepted as representing emmer. They are accompanied by free-threshing wheats, broomcorn millet, and foxtail millet, although in comparatively modest quantities. Furthermore, occasionally einkorn and possibly rye and oat have been encountered, but whether these were actively exploited or rather have entered the assemblage as weeds is currently not possible to ascertain.

Beyond cereals, several taxa of pulses, fruits, and nuts have been identified. Broad bean is most abundant, followed by lentils and bitter vetch. It is likely that a variety of other pulses were exploited, including peas and vetchlings, but, for these seeds, morphology is often overlapping and fragmentation is quite common, so that none of these could so far be securely identified to species-level. Amongst the fruits and nuts grapevine, fig, elderberry almond, and possibly pistachio, have been recovered from the main phases, while olive and apple derive from so far unstratified deposits.

All in all, even when uneven preservation rates in the charred record are considered, it is likely that cereals, especially emmer and barley, throughout all phases constituted the main staple of the plant-based diet, supplemented by minor amounts of other wheats and millets, and accompanied by a variety of pulses including at least broad bean, lentils, and bitter vetch. This exploitation pattern remains stable throughout at least the first centuries of Tarquinia's habitation with only minor fluctuations visible, that beyond actual past change as well simply might reflect variation in the quality of the data. The primary cultivation of the more stress-tolerant species barley, emmer, and broad bean is an especially common phenomenon of the region, but also beyond, and suggests a focus on reliable staples to ensure a solid food supply during the formation of the city.

Only a limited number of fruit and nut taxa have so far been identified, while other common foods, such as vegetables, herbs, and spices are completely absent. This is a common bias in the charred record, where various taphonomic processes favour the preservation of only specific aspects of the exploited plant spectra, and hence the original diet would have been much more diverse than reflected in the assemblage presented here.

**Zooarchaeological analyses**

The study of animal remains from the ‘monumental complex’ at Tarquinia revealed an intensification in animal exploitation through the millennium, with an increasingly controlled management and several changes in product-production focus. The Villanovan period was characterised by unspecialised animal management: the age-at-death estimation analyses of the three main animals, cattle, caprines and pigs, did not show a preferred slaughtering age, with animals killed at a young, subadult and adult stage. This strategy might indicate that animal product production was on a small scale and that it was not subjected to a centralised authority. Data from the Orientalising period is limited by the scarcity of animal bones dated to this period. Despite this, data suggest that since the Orientalising period, the small-scale unspecialised management was transformed into a more regulated, product-focused economy, with pigs replacing caprines as the preferred animals, although the rapidity and the ways in which it evolved are unclear. As a result, data show that during the archaic period mainly adult cattle and caprines were culled and a more complex organisation of pig rearing was implemented, with controlled breeding and slaughtering seasons. Analyses suggested a more widespread use of cattle for traction and an enhanced interest in wool production, which also indicates an increasingly regulated use of animal resources. Such transformations occurred in a period of great social and cultural changes at Tarquinia, including demographic growth, increasing social stratification and urbanization, with a more organised control over the rural landscape. Along with such transformations, faunal data clearly show that animal management underwent profound changes to support the dietary and economic needs of the population. During the Hellenistic period, a second important change occurred in animal management, when the variety of species consumed increased enormously, and caprine and pig breeding switched its focus to younger animals for the production of high-quality pork and lamb meat and caprine dairy products. The changing patterns above described were also observed in many other central Italian Tyrrhenian sites, which indicates that the evolution of cattle and caprine management and exploitation detected at Tarquinia was part of a regional trend.

Unique traits of the ‘monumental complex’ assemblage are related to the religious nature of the site that resulted in uncommon patterns, especially during the Villanovan period, with an exceptional number of cranial elements of caprines, pigs and dogs collected in several types of contexts (huts, pits, fireplaces). In addition, hundreds of red deer antler fragments were collected in area alpha (see above) and its surroundings.

**Dating methodology and isotope analysis for palaeodietary reconstruction methods (Figure S3**; **Tables S1 and S2)**

The main radiocarbon (^14^C) measurements were performed using AMS at the 14CHRONO Centre, Queen’s University Belfast, using established protocols^54^. For tooth roots (Supplementary Tables S1, S2), δ^13^C and δ^15^N isotope values were determined on a separate line using isotope ratio mass spectrometry (IRMS). This allowed us to exclude any contaminated samples (none were found), and evaluate the palaeodietary sources of the proteins dated in the sample via AMS. Marine diet influences the radiocarbon age of the sample, and must be investigated before the ^14^C measurements can be correctly age calibrated.

Signals of marine diet were investigated through comparison of the IRMS results with published data, and through Bayesian mixture modelling. This provides a formal way to estimate the amount of marine-derived protein in each radiocarbon sample. The R software package simmr^55^ was used to undertake this analysis using the trophic enrichment factors for bone collagen of 0.8 ± 0.5 ‰ for ^13^C and 4 ± 1 ‰ for ^15^N. A ^13^C offset

of 4.8 ± 0.5 ‰ was used for seed-to-collagen sources. Baseline measurements for dietary sources were obtained from samples of animal bones and carbonated seeds (Table S2B). These were supplemented from published literature estimating a baseline of -13.5±2.1 ‰ for δ^13^C and 8.4 ±1.2 ‰ for δ^15^N for marine collagen samples^56^.

Each ^14^C sample was then age calibrated using a bespoke mixture of the terrestrial northern hemisphere IntCal20^57^ and the Marine20^58^ datasets, with the degree of mixing dependent on the Bayesian estimate of the proportion of marine protein in each sample.

Bayesian analysis was also used to include information about the relative chronological order (Figure S3), which could be identified through stratigraphic knowledge in the case of the Civita stratigraphy. Skeletons of two individuals (11 and 12) were found in direct association with distinctive and relatively commonplace Early Iron Age fibulae known to date before 750 BC, a date established by typology and cross-dating. This information was included as an age constraint on the ^14^C samples from these skeletons. OxCal 4.3^59^ was used to specify this information and undertake a full Bayesian analysis using Markov Chain Monte Carlo methods. This allowed us to determine whether the specification of chronological information, including the estimates for marine carbon, was consistent with how their relationships were specified and constrained by archaeological information. Alternative specifications of the chronological information were also explored to determine whether the analysis was sensitive to the specification of prior information, both with and without the archaeological constraints, producing consistent results albeit with greater uncertainty. The OxCal code used to specify the preferred model is given below in the section ‘Supplementary Code’.

**Oxygen (δ^18^O) isotope analysis methods (Table S3)**

δ^18^O was undertaken on the structural carbonate of bioapatite (δ^18^O), since it is cost effective compared with the oxygen isotope values extracted from phosphate (δ^18^O). Following abrasion of the external crown enamel surface using a Dremel rotary tool, enamel powder of approximately 8–12 mg was drilled out from the entire crown height avoiding any adhering dentine. Enamel powders were then treated for bioapatite extraction using established methods^60^ After soaking samples in 2–3% aq. sodium hypochlorite (24 hours at 4°C) to remove organic matter, the samples were rinsed five times in distilled water and mixed with 0.1 M acetic acid (0.1 ml/mg) for four hours at room temperature to remove exogenous carbonate. Following five rinses, the samples were placed in the freezer at -20°C for 1 hour, and then at -80°C for an additional 30 minutes before being freeze dried for 90 minutes. The dried samples were then transferred into suitable tubes and placed into a VG SIRA mass spectrometer, where each sample was reacted with 100% ortho phosphoric acid at 70°C. Liberated CO2 was then trapped and transferred to the mass spectrometer for the isotopic analysis. Results are reported with reference to the international standard VPDB calibrated through the NBS19 standard ^61, 62^ and the long-term analytical precision is better than ±0.08‰ for ^13^C/^12^C and better than ±0.10‰ for ^18^O/^16^O.

**Strontium (^87^Sr/^86^Sr) isotope analysis methods (Table S3)**

**^87^Sr/^86^Sr isotope analysis sampling**

As for inhumations, tooth enamel was sampled following a previously published protocol^63^. A flexible diamond-edged rotary wheel mounted on a drilling machine (DREMEL® model 300) was used to cut a crown section of the cusps. Adhering contaminants such as soil, sediments and all traces of dentine were removed using a dental bur. We aimed at obtaining a sample mass for Sr isotopic analysis of enamel between 0.02-0.04 g.

The petrous bone was sampled following the VUB (Vrije Universiteit Brussel) protocol^64^ to ensure precise sampling of the otic capsule, which does not remodel after development, thus providing a temporally resolved early life signal. Each petrous bone was externally abraded with a rotary diamond bur to remove adhering contaminants. The samples were subsequently cut in half across the internal auditory meatus using a Strong 209 precision drill with diamond wheel attachment. This process creates a midmodiolar section, leaving the cochlea, the vestibulum, and semicircular canals visible. The exposed surfaces were further mechanically cleaned with a dental bur to reduce contamination. All equipment was thoroughly cleaned between each sampling to prevent cross-contamination. The petrous bones, cut in halves, were inserted in pre-cleaned and pre-labelled glass test-tubes and pre-treated with a three steps cleaning procedure^65^ before sampling the bone powder. The procedure consisted of: (1) cleaning samples with Milli-Q® water. Samples were immersed in Milli-Q® water and left in ultrasonic bath for 10 min. Glass test tubes were emptied and samples rinsed with Milli-Q® two more times and filled up again with Milli-Q®. Each test tube was kept for roughly 1 min in the ultrasonic bath until appearance of dirt ceased. Samples were rinsed twice again Milli-Q® and Milli-Q® water discharged. All the procedure was repeated two more times. (2) Samples were further cleaned with acetic acid (CH_3_COOH). The test tubes were filled up with 1 M acetic acid and left for 7 min in the ultrasonic bath. Subsequently, each test tube was kept for roughly 1 min in the ultrasonic bath until the solution was clear. Samples were rinsed twice again with Milli-Q® and Milli-Q® water discharged. Step (1) was repeated. The cleaned petrous bones samples were dried overnight in an oven at 50°C. The internal cortex of the otic capsule was sampled with a cleaned diamond-tipped bur and stored in pre-cleaned plastic Eppendorf (1.5 ml) vials. Cremated teeth followed the same cleaning pre-treatment. The bone powder mass ranged between 0.01 and 0.02 g. We aimed at obtaining a sample mass for Sr isotopic analysis of cremated enamel between 0.02-0.04 g. We aimed at obtaining ~2 g for soil, ~1.5 g for seeds and 0.02-0.04 g for fauna enamel samples.

**^87^Sr/^86^Sr isotope analysis laboratory procedure: baseline samples, tooth enamel and petrous bones powder samples (Figure S4) (Table S3)**

Sample preparation: soil and seed samples and (inhumed) human and fauna tooth enamel from the Civita were analysed at the Frankfurt Isotope and Element Research Center (FIERCE) following established protocols reported in ^63, 66^. Cremated teeth and petrous bones from Tarquinia Villa Bruschi were analysed at Cardiff Earth Laboratory for Trace element and Isotope Chemistry (CELTIC). Samples were transferred in pre-cleaned Teflon™ beakers. 1 ml of 14M HNO_3_ was added to the sample and left for two days at 120° on hotplate. Samples were subsequently dried down on the hot plate at 120°and then 8M HNO_3_ were added to the beakers. Samples in 8M HNO_3_ were put into the ultrasonic bath for 45 min and left overnight on a hot plate. Samples were then transferred from the beakers into pre-cleaned microcentrifuge tubes and left in the centrifuge for c. 3 min. Strontium separation and mass spectrometry followed established practice^67^. Sr from all samples was extracted using SP columns, pre-cleaned in 5-10% HNO_3_ solution for ~48 h. Columns were filled with c. 100 µL of pre-cleaned Sr Spec™ resin using a revised version of the protocol^68^ and further cleaned with 0.05M HNO_3_. After sample introduction in 8M HNO_3_, 1300 μl of 8M HNO_3_ was added into each column to remove matrix elements (including Ca and traces of Rb) followed by 1500 μl 0.05M HNO_3_ to collect the Sr fraction. This step was repeated a second time to ensure optimal removal of Ca and Rb impurities from the Sr cut.

Mass spectrometry: Sr isotopic analysis for tooth human enamel (inhumed) and baseline samples was undertaken at the Frankfurt Isotope and Element Research Center (FIERCE). ^87^Sr/^86^Sr ratios were measured in static mode using a NeptunePlus™ Multicollector-ICPMS at the Frankfurt Isotope and Element Research Center (FIERCE) of Goethe University^69^. The reproducibility of the Sr isotopic standard SRM987 during the analytical session yielded a ^87^Sr/^86^Sr value of 0.710271 ± 0.000020 (2 SD, n=6); as this is within error of the accepted value of 0.710248^70^ (and its uncertainty), no (minor) adjustment of obtained ratios was performed. Sr blank measurements were conducted using a dilute 84Sr-enriched tracer solution (“spike”) and found to be negligible relative to the relatively large Sr samples processed. The errors (at 95% c.l.) of the individual samples analysed at FIERCE and reported in Supplementary Table S3 represent the quadratically propagated SRM987 uncertainty (2 SD) combined with the in-run errors of each individual sample analysis (2 SE). The individuals from Tarquinia Villa Bruschi were sampled at the CUBA laboratory (Cardiff University BioArchaeology) and the ^87^Sr/^86^Sr ratios analysed at Cardiff Earth Laboratory for Trace element and Isotope Chemistry (CELTIC) using a Nu Plasma II multi-collector inductively coupled plasma mass spectrometer (MC-ICP-MS). Samples were introduced using an Aridus II desolvator introduction system. All data were first corrected for on-peak blank intensities, then mass bias corrected using the exponential law and a normalization ratio of 8.375209 for ^88^Sr/^86^Sr^71^. Residual krypton (Kr) and rubidium (^87^Rb) interferences were monitored and corrected for using ^82^Kr and ^83^Kr (^83^Kr/^84^Kr = 0.20175 and ^83^Kr/^86^Kr = 0.66474; without normalization) and ^85^Rb (^85^Rb/^87^Rb = 2.5926), respectively. Analysis of NIST SRM 987 during the analytical session gave a ^87^Sr/^86^Sr value of 0.710292 ± 0.000007 (2*σ*_N_, *n* = 11), and all data are corrected to NIST SRM 987 values of 0.710248^72^. Total procedural blanks are typically less than 20pg of Sr, which is negligible relative to the Sr in samples (greater than 20 ng). Accuracy of the NIST SRM 987 normalization and the chemistry processing was assessed by repeat measurements of ^87^Sr/^86^Sr ratio in NIST SRM 1400 (Bone Ash, processed through chemistry similar to the unknown samples), giving an average ^87^Sr/^86^Sr ratio of 0.713111 ± 0.000014 (2*σ*_N_, *n* = 5), which is consistent with the published value (0.713126 ± 0.000017^73^).

**aDNA methods (Tables S4-12)**

**DNA extraction**

Sample processing was carried out in a dedicated ancient DNA facility in Trinity College, Dublin, Ireland. All bones were photographed extensively prior to further processing and UV-irradiated for 15 minutes on either side. A section of the otic capsule region was removed and pulverised. An aliquot of ~100mg of the bone powder was subjected to a silica column method^74^, with modifications^75^, with an initial washing step by 0.5% bleach solution and by pre-digestion solution^76^. The digestion step contained a further modification of a single incubation at 37 °C for 24 h. DNA extracts were purified with silica columns (MinElute PCR purification kit, Qiagen, Hilden, Germany) and eluted in 55 μL of Elution Buffer.

**Library preparation and sequencing**

An aliquot of each extract was used to construct a double-stranded DNA NGS library treated with Uracil-DNA-glycosylase (UDG) prior to the library construction using an established method^77^ with modifications^78^, with the exception of the initial screening libraries which were not treated with UDG to assess damage patterns. The screening libraries were sequenced on the NovaSeq Illumina platform (S1 flow cell, 50-bp paired-end) at Trinseq, St. James’ Hospital, Dublin. Damage (deamination) patterns and fragment length distributions were assessed post-alignment and processing using mapDamage2.0^79^. All samples showed damage and fragmentation patterns consistent with ancient DNA.

DNA extracts with >10% human endogenous content were selected for higher coverage sequencing (Supplementary Tables S4 and S7). Between 4 and 10 PCRs per sample with unique double-index combinations were prepared from each UDG-treated library to increase complexity. The concentration and quality were then assessed using the Agilent Tapestation 2200 system with a D1000 screentape. The sequencing was performed at Source Bioscience, UK on the NovaSeq Illumina platform (S4 flow cell, 150-bp paired-end). Further details of sequencing and bioinformatic processing results can be found in Supplementary Table S7.

**Bioinformatic sample processing**

AdapterRemoval v2^80^ was used to remove sequencing adapters from raw FASTQ files, and reads were collapsed if the overlap between read pairs was ≥11 bp. Trimmed FASTQs were aligned to the human reference genome (hs37d5) using bwa aln^81^ with relaxed parameters (-o 2, -n 0.01) with seeding disabled. Alignment .sai files were converted to BAM format and read groups added for each amplified library using BWA sampe (for uncollapsed pairs), or BWA samse (for collapsed reads)^81^. Samtools was used to sort the aligned files by chromosomal position^82^. These files were merged to sample level within each sequencing run using Picard’s MergeSamFiles tool^83^. PCR and optical duplicates were removed using the Picard MarkDuplicates tool. Merges to sample level were then carried out across sequencing runs, with Picard MergeSamFiles. Indels were realigned using GATK^84^. A mapping quality filter of ≥25 was applied using samtools. To mitigate reference bias a read length filter of ≥34bp was applied. The terminal 2 bases on both the 3’ and 5’ ends of all reads were soft-clipped (PHRED score reduced to 2). Genomic coverage was estimated using Qualimap v2^85^ with default parameters.

**Molecular sexing**

Molecular sexing was carried out using established methods^86^. The results are given in Supplementary Table S4.

**Uniparental analyses: Mitochondrial haplogroups and contamination**

For mitochondrial analyses the trimmed FASTQ files were realigned to the revised Cambridge Reference Sequence (rCRS, NC_012920.1), with a mapping quality filter ≥30 and all other filters as above. Haploid genotypes for the entire mitochondrial genome were called using bcftools^87^, filtered to include only sites covered by at least three reads, and the VCF file was inputted into a local version of HaploGrep2^88^, for haplogroup determination, which uses the Phylotree build 17 nomenclature^89^. For haplogroup assignment and contamination analyses indels and known mutational hotspots were excluded.

Mitochondrial contamination levels were estimated by calculating the proportion of all polymorphic sites with >1 alleles called (with known mutational hotspots excluded), and the calculation was repeated excluding sites which could be the result of post-mortem damage (transitions). Mitochondrial genome coverage was estimated using Qualimap v2^85^ with default parameters.

The haplogroup assignments and contamination estimates are given in Supplementary Table S4. The full results of the haplogroup assignments are given in Supplementary Table S8.

**Uniparental analyses: Y-chromosome haplogroups (Tables S4, S5)**

Y-chromosome haplogroups were assigned using the Y-chromosome haplogroup marker SNPs from the ISOGG Y-chromosome database [(Version 15.73, July 2020).](https://paperpile.com/c/4MPIdA/ge37f)^90^ The base calls for all positions were piled up with GATK and filtered to retain only calls with base quality ≥30. For each major Y-chromosome lineage any loci with base calls for the derived allele for were extracted and manually examined to determine Y-chromosome haplogroups. As the male samples were sequenced to a depth of 0.86 and 1.7X genomic coverage there was low coverage over most informative Y-chromosome haplogroup markers, and some were missing. For this reason, the possibility of the individuals belonging to a slightly more derived haplogroup cannot be excluded. Haplogroup information can be found in Supplementary Table S4, and the precise haplogroup assignation is explored in more detail in Supplementary Table S9.

X-chromosome contamination in male individuals was estimated using ANGSD, with transition SNPs removed to exclude the effects of post-mortem molecular damage, following published methods ^91,92^. The contamination estimates are given in Supplementary Table S4.

**Pseudohaploid analyses**

Calling pseudohaploid genotypes from ancient samples is a well-established method to circumnavigate the issue of low genomic coverage in ancient samples. Pseudohaploid genotypes (filtered for base quality ≥30) were called for all autosomal SNPs (~1.15 million) from the “1240K” SNP panel^93^ usings previously established methods^94^ in a number of published individuals^93,95-126^ (a full list of published individuals and their inclusion in analyses can be found in Supplementary Table S10). Previously published individuals from Italy^119^ were only considered for inclusion in the dataset if directly dated, or dated by kinship.

For reference population groupings see Supplementary Table S10. Population abbreviations include: BB (Bell Beaker); BA, LBA (Bronze Age, Late Bronze Age); IA (Iron Age).

**Principal component analysis (PCA)**

For PCA the final assembled dataset of published ancient individuals was filtered to remove relatives, individuals with noted contamination (e.g. mtDNA estimates >5%), and some individuals who are extreme outliers to the rest of their group. The dataset was filtered to retain approximately 595,000 SNPs from the 1240K SNP set which overlap with the Human Origins array^111^. Only individuals with ≥100,000 SNPs overlapping with the Human Origins array were retained for the analysis. The ancient dataset was merged with a dataset of 602 individuals from 71 European and West Asian populations from the Human Origins dataset^111^. The genetic variation of the ancient individuals was then projected onto the first two principal components of the variation found in the modern individuals with smartpca v13050, part of the EIGENSOFT toolset ^127, 128^ using the option “lsqproject: YES”, with SNPs removed if the correlation between them was >0.2 (r2thresh: 0.2). The resulting plots in Figure 4 were constructed using the *ggplot2* package ~~as part of~~ in R.

**qpAdm Admixture Modelling**

qpAdm, based on *f4*-statistics^129^, was used to estimate the ancestry proportions (summed to 1) for the new Iron Age individuals, broadly following the established approach. The dataset used was the autosomal “1240K” SNPs called in the new and published ancient individuals. Details of the published ancient individuals included in each population can be found in Supplementary Table S10. In general, the model was considered a good fit if the p-value for the model was ≥0.05, and marginally acceptable if the p-value was ≥0.01. Models were rejected if the p-value was <0.01, or if a negative admixture fraction was reported for any source population in the model as this implies the model is infeasible. A stable set of “right” populations as previously used for similar populations^96^ was used, and populations were removed from this set if they were instead used as a “left” source population.

*Right populations:* Mota (n=1), Ust’Ishim (n=1), Kostenki14 (n=1), MA1 (n=1), Natufian (n=6), Vestonice16 (n=1), Villabruna (n=1), Anatolian_N (n=26), CHG (n=2), EHG (n=3), GoyetQ116-1 (n=1), Jordan PPNB (n=2), Iran_N (n=9), ElMiron (n=1), Morocco_Iberomaurusian (n=7), WHG (n=6), YamnayaSamara (n=9).

All tests were run with the following options; “INBREED: YES” as recommended for any run with pseudohaploid data, and “ALLSNPS: YES” which uses the maximum number of overlapping SNPs for each *f*-statistic. If a population had <50,000 SNPs it was not considered for any model.

Individuals from Tarquinia were modelled as a 2-way mixture of the Italy_Beaker (I1979) and YamnayaSamara (Steppe-related ancestry) (Model 1) as an initial 1-way model using just Italy_Beaker as a single source was not a good fit for most of the Tarquinian individuals (Supplemental Table S11).

For the outlier Individual 11, a number of 1-way models were run (Model 2). The source populations tested were late Bronze Age or Iron Age European populations.

*Potential source populations:* Armenia_LBA, Britain_IA, Bulgaria_IA, Croatia_LBA_EIA, Denmark_IA, Estonia_IA_tarand, Etr_CAM_Tus_CEU, Etr_late_VET_CEU, France_IA_East, France_IA_North, France_IA_South, Iran_IA, Ireland_BA, Norway_IA, Russia_IA_Scythian, Sardinia_PhoenicianPunic, Scotland_IA, Scotland_IA_Pict, Spain_East_IA, Spain_North_IA, Spain_SWest_IA, Sweden_IA, Unetice_Poland_BA.

Accepted models are given in Supplementary Table S5, and full details of all models can be found in Supplementary Table S11.

**Imputation**

Imputation of diploid genotypes was carried out on the new individuals from Tarquinia and the previously published shotgun sequenced individuals from the Iron Age to Late Antiquity^96^. 77.8 million biallelic SNPs from the 1000 Genomes panel^130^ were called in all samples using bcftools^87^. The software GLIMPSE^131^ was used to impute missing diploid genotypes in each individual singly to avoid potential batch effects, using the 1000 Genomes reference panel. Post-imputation the individuals were merged into a single dataset. The resulting genotypes were then filtered to retain only genotypes with genotype probability ≥66% (GP66) and ≥99% (GP99).

**Pigmentation Phenotype Prediction (Table S6)**

The GP66 imputed dataset was filtered to retain only the pre-Imperial Roman Iron Age Italian individuals, and genotype calls for the 41 h-Irisplex-S SNPs were extracted. The calls were converted to h-Irisplex-S coding and uploaded to the web server for phenotype predictions. The pigmentation profiles for each individual were determined using established rubrics ^132,133,134^. The results are displayed in Supplementary Table S6, and the complete output of the h-Irisplex-S model can be found in Supplementary Table S12.

**Kinship Analysis**

Kinship analysis was first performed using the pseudohaploid dataset. The newly reported individuals from Tarquinia and published individuals from the Iron Age to Late Antiquity from mainland Italy^96^ were extracted from the larger dataset. The analysis was performed using READ^135^ with default parameters. The results did not indicate any first or second degree kinship between the newly reported individuals, nor with any of the published individuals. As READ lacks the power to detect relationships beyond the second degree a second kinship analysis was carried out using the same set of individuals, this time using the imputed diploid genotypes (GP99 dataset). The dataset was filtered to retain only transversions with a global minor allele frequency of ≥5% in the 1000 Genomes Phase 3 panel, for a final 2.21 million SNPs. The analysis was performed using the software KING^136^ (v.2.2.4) with the option “--related”. This software can detect relationships up to the 4^th^ degree, and again no kinship was reported between the new individuals in this study.

# **Supplementary figures**

Figure S1 – Position of Tarquinia on the recently published isoscape map by Lugli and colleagues^16^. The local bioavailable ^87^Sr/^86^Sr values for Tarquinia are 0.7091-0.7094.

| 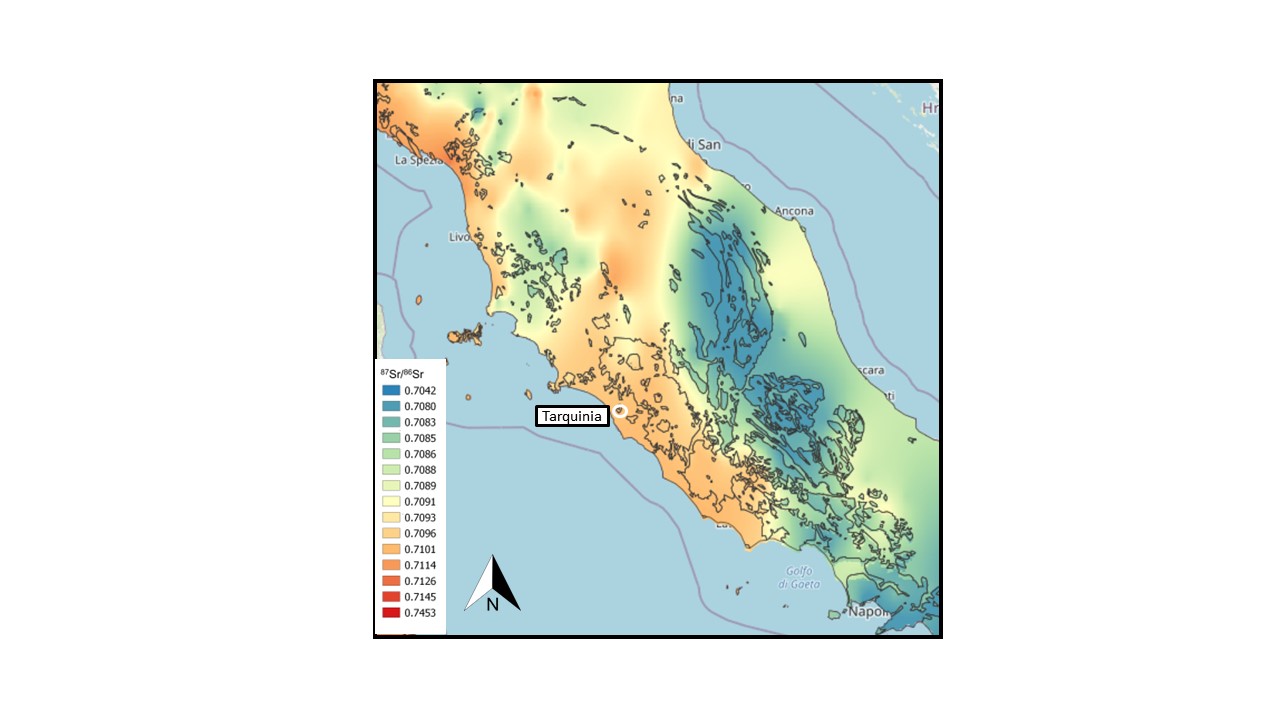 |
| --- |
|  |

Figure S2 Simplified sketch of a cross-section of the sedimentary deposits constituting the Tarquinia Civita^15^. 1) Early Pliocene mudstones ‘Argille Azzurre Formation’; 2) Middle-Late Pliocene calcarenite and limestone, ‘Macco Unit’; 3) Late Pliocene-Early Pleistocene mudstone and sandstones ‘Pian della Regina Unit’; 4) Middle Pleistocene (?), Terra Rossa-like palaeosol; 5) Late Holocene archaeological deposits.


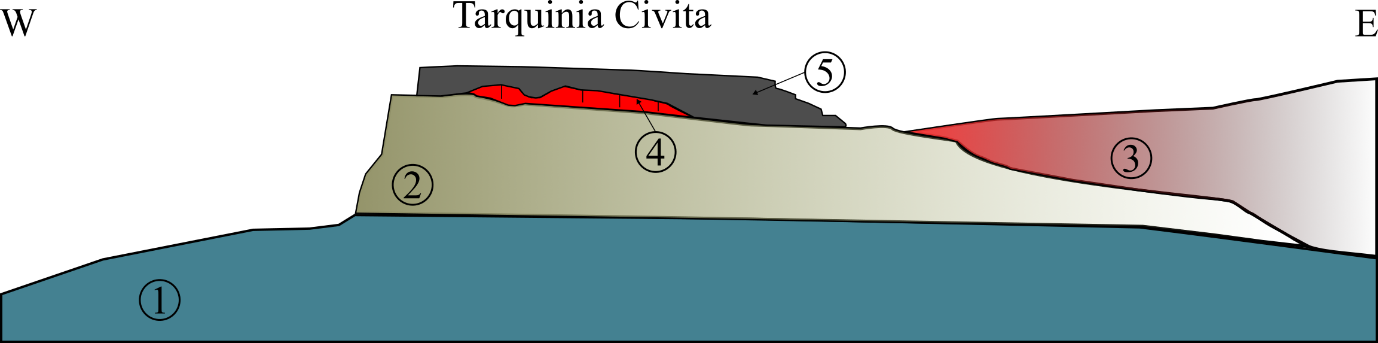


| Figure S3– Results posterior distributions of the radiocarbon dates in the chronological model described in the Supplementary Code below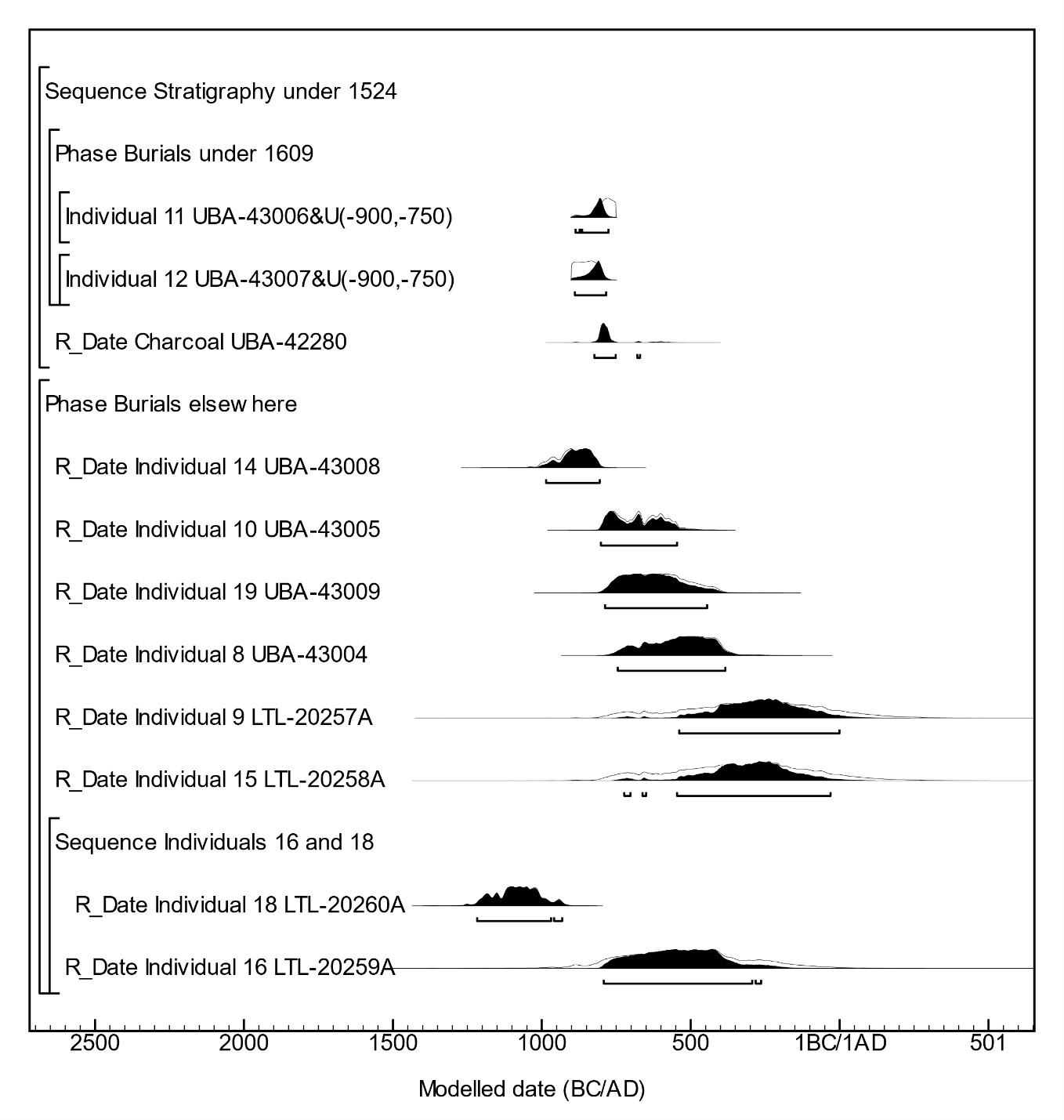 |
| --- |

Figure S4 – Box and whisker plots with ^87^Sr/^86^Sr baseline values for Tarquinia. Seed and soil come from the area around the Civita. Archaeological fauna was collected at the Civita. YC = young children (1-5 years of age) from Tarquinia Villa Bruschi. OC = older children (5-10 years of age) from Tarquinia Villa Bruschi.

| 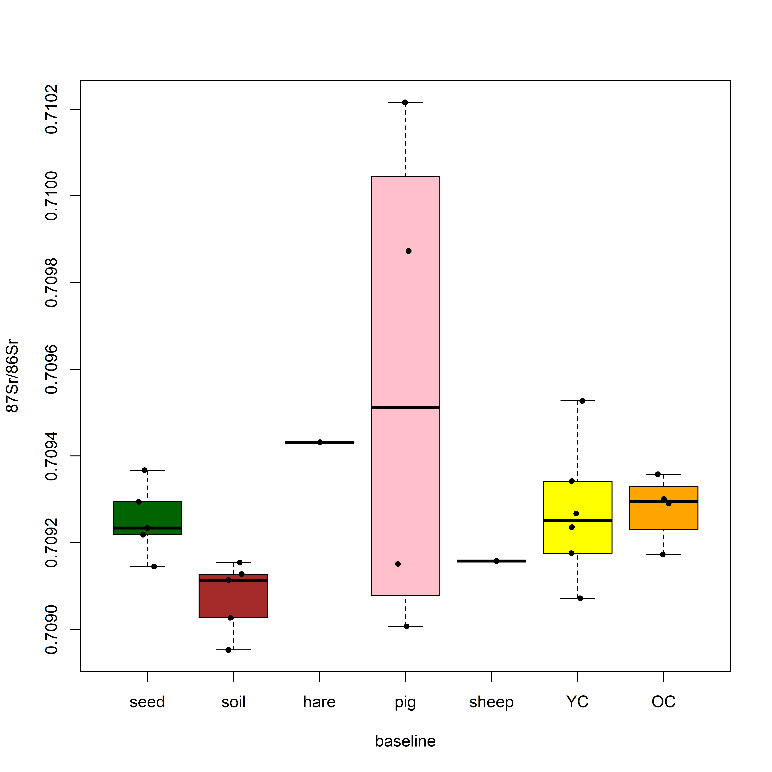 |
| --- |

**SUPPLEMENTARY TABLES**

Table S1 – Radiocarbon dates from tooth roots and bones from ten individuals from Tarquinia Civita and a charcoal sample.

A. Belfast dates employed in the dietary modelling

| **Lab. ID** | **Sample** | **^14^C date** | **Context** | **Unmodelled calibrated date BC** | | **Modelled calibrated date BC** | |
| --- | --- | --- | --- | --- | --- | --- | --- |
|  |  |  |  | **0.68 prob.** | **0.95 prob.** | **0.68 prob.** | **0.95 prob.** |
| UBA-43004 | ULM1/root | 2620±35 | Individual 8 | 660–400 | 750–380 | 660–400 | 740–380 |
| UBA-43005 | LRM2/root | 2618±32 | Individual 10 | 790–570 | 800–540 | 790–580 | 800–540 |
| UBA-43006 | LRM2/root | 2747±36 | Individual 11 | 820–660 | 890–540 | 830–790 | 890–770 |
| UBA-43007 | LRM2/root | 2792±40 | Individual 12 | 900–810 | 990–790 | 840–790 | 900–780 |
| UBA-43008 | LRM2/root | 2828±28 | Individual 14 | 930–830 | 1000–810 | 920–830 | 980–810 |
| UBA-43009 | LRM2/root | 2709±24 | Individual 19 | 740–540 | 780–420 | 740–550 | 780–430 |
| UBA-42280 | charcoal | 2602±36 | Context 1524 | 810–780 | 830–590 | 800–780 | 820–600 |

B. Dates from Lecce from skeletal remains of other individuals buried at the site

| **Lab. ID** | **Sample** | **^14^C date** | **Context** | **Calibrated date BC** | |
| --- | --- | --- | --- | --- | --- |
|  |  |  |  | **0.68 prob.** | **0.95 prob.** |
| LTL-20257A | Humerus (4gr) | 2387±45 | Individual 9 | 750-390 | 750-390 |
| LTL-20258A | petrous (intera) | 2419±45 | Individual 15 | 750-400 | 750-400 |
| LTL-20259A | tibia (3.6gr) | 2578±45 | Individual 16 | 820-550 | 820-550 |
| LTL-20260A | Parietal (1.6gr) | 2892±45 | Individual 18 | 1190–1010 | 1220–930 |

Table S2 – Stable carbon and nitrogen results.

A Human remains

|  | **δ^15^N (AIR)** **‰** | **δ^13^C (VPDB) ‰** | **C:N ratio** | **Mixture model estimates** | | |
| --- | --- | --- | --- | --- | --- | --- |
|  |  |  |  | **marine** | **cereals** | **animals** |
| Individual 14 | 9.2 | -18.7 | 3.18 | 0.26±0.09 | 0.35±0.17 | 0.4±0.22 |
| Individual 12 | 9.2 | -19.6 | 3.17 | 0.19±0.1 | 0.42±0.19 | 0.39±0.22 |
| Individual 11 | 9.9 | -16.3 | 3.15 | 0.49±0.12 | 0.23±0.12 | 0.28±0.16 |
| Individual 19 | 10.3 | -13.8 | 3.16 | 0.69±0.1 | 0.15±0.08 | 0.16±0.1 |
| Individual 8 | 8.6 | -14.2 | 3.17 | 0.64±0.11 | 0.19±0.11 | 0.17±0.12 |
| Individual 10 | 8.6 | -18.6 | 3.16 | 0.25±0.1 | 0.37±0.18 | 0.38±0.22 |

B: Domestic animals and cereals, used as baseline in mixture models

| **Lab ID** | **Species** | **δ^15^N (AIR) ‰** | **δ^13^C (VPDB) ‰** | **C:N ratio** |
| --- | --- | --- | --- | --- |
| UB-43010 | sheep | 8.53 ± 0.29 | -20.37 ± 0.36 | 3.20 |
| UB-43013 | pig | 3.35 ± 0.29 | -20.83 ± 0.36 | 3.21 |
| UB-43014 | pig | 6.39 ± 0.29 | -20.39 ± 0.36 | 3.20 |
| UB-43017 | hare | 5.33 ± 0.29 | -22.19 ± 0.36 | 3.19 |
| UB-43018 | domestic | 4.96 ± 0.29 | -18.99 ± 0.36 | 3.18 |
| UB-43019 | domestic | 4.05 ± 0.29 | -20.71 ± 0.36 | 3.18 |
| Average of animal bone measurements excluding outliers (mean ± s.d.) | | 5.7 ± 1.7 | -20.6 ± 1.2 | N/A |
| Average of 145 measurements from individual cereal grains (mean ± s.d.) | | 2.9 ± 1.5 | -22.6 ± 1 | N/A |

Table S3 – δ^18^O and ^87^Sr/^86^Sr for baseline and human samples. Errors (at 95% c.l.) of the individual samples analysed at FIERCE represent the quadratically propagated SRM987 uncertainty (2 SD) combined with the in-run errors of each individual sample analysis (2 SE). For the samples analysed in Cardiff, error represent the internal precision for each measurement.

| \| **n** \| **Id** \| **Site** \| **Chron** \| **Geo-lithology** \| **Sex/Age** \| **Type of sample** \| **δ^13^C_ca_ V-PDB**  **(‰)** \| **±** \| **δ^18^O_ca_ V-PDB (‰)** \| **±** \| **δ^18^O_ca_ V-SMOW**(**‰**) \| **δ^18^O_p_ V-SMOW**(**‰**) \| ^87^Sr/^86^Sr \| (±95% c.l.) \| \| --- \| --- \| --- \| --- \| --- \| --- \| --- \| --- \| --- \| --- \| --- \| --- \| --- \| --- \| --- \| \| 1 \| Tr-Cv-8 \| Tarq_Civ \| arch \| - \| F/AD \| homo_ULM1 \| -8.37 \| 0.01 \| -5.62 \| 0.03 \| 25.07 \| 16.19 \| 0.711150 \| 0.000031 \| \| 2 \| Tr-Cv-10 \| Tarq_Civ \| arch \| - \| M/AD \| homo_LRM2 \| -12.04 \| 0.02 \| -3.17 \| 0.04 \| 27.60 \| 18.80 \| 0.708878 \| 0.000024 \| \| 3 \| Tr-Cv-11 \| Tarq_Civ \| arch \| - \| F/MAT \| homo_ LRM2 \| -6.90 \| 0.02 \| -6.22 \| 0.04 \| 24.45 \| 15.55 \| 0.712534 \| 0.000028 \| \| 4 \| Tr-Cv-12 \| Tarq_Civ \| arch \| - \| F/AD \| homo_ LRM2 \| -13.31 \| 0.02 \| -4.60 \| 0.03 \| 26.12 \| 17.27 \| 0.708922 \| 0.000023 \| \| 5 \| Tr-Cv-14 \| Tarq_Civ \| arch \| - \| F/AD \| homo_LLM2 \| -13.27 \| 0.01 \| -2.17 \| 0.03 \| 28.62 \| 19.86 \| 0.708955 \| 0.000029 \| \| 6 \| Tr-Cv-19 \| Tarq_Civ \| arch \| - \| M/IND \| homo_ LRM2 \| -8.78 \| 0.02 \| -4.81 \| 0.03 \| 25.90 \| 17.05 \| 0.708992 \| 0.000031 \| \| 7 \| Tq-Bs-1 \| Pian_ Reg \| arch \| - \| - \| soil \| - \| - \| - \| - \| - \| - \| 0.709154 \| 0.000023 \| \| 8 \| Tq-Bs-2 \| Pian_ Reg \| modern \| Clayish-sand calcarenite \| - \| seed \| - \| - \| - \| - \| - \| - \| 0.709234 \| 0.000035 \| \| 9 \| Tq-Bs-5 \| Pian_ Civ \| modern \| Macco \| - \| soil \| - \| - \| - \| - \| - \| - \| 0.708953 \| 0.000022 \| \| 10 \| Tq-Bs-6 \| Pian_ Civ \| modern \| Macco \| - \| seed \| - \| - \| - \| - \| - \| - \| 0.709294 \| 0.000031 \| \| 11 \| Tq-Bs-7 \| Pian_ Civ \| modern \| Middle and Upper Plioc \| - \| soil \| - \| - \| - \| - \| - \| - \| 0.709128 \| 0.000023 \| \| 12 \| Tq-Bs-8 \| Pian_ Civ \| modern \| Middle and Upper Plioc \| - \| seed \| - \| - \| - \| - \| - \| - \| 0.709367 \| 0.000027 \| \| 13 \| Tq-Bs-9 \| South_ Poggio_Sorg \| modern \| Clay (?) Lower Pleist \| - \| soil \| - \| - \| - \| - \| - \| - \| 0.709114 \| 0.000022 \| \| 14 \| Tq-Bs-10 \| South_ Poggio_Sorg \| modern \| Clay (??) Lower Pleist \| - \| seed \| - \| - \| - \| - \| - \| - \| 0.709145 \| 0.000029 \| \| 15 \| Tq-Bs-13 \| Fosso_S_Savino \| modern \| Marine grey clay. Lower Plioc \| - \| soil \| - \| - \| - \| - \| - \| - \| 0.709027 \| 0.000023 \| \| 16 \| Tq-Bs-14 \| Fosso_S_Savino \| modern \| Marine grey clay. Lower Plioc \| - \| seed \| - \| - \| - \| - \| - \| - \| 0.709218 \| 0.000029 \| \| 17 \| Tq-Bs-15 \| Tarq_Comp_Mon \| arch \| - \| - \| sheep_enamel \| - \| - \| - \| - \| - \| - \| 0.709158 \| 0.000024 \| \| 18 \| Tq-Bs-17 \| Tarq_Comp_Mon \| arch \| - \| - \| pig_enamel \| - \| - \| - \| - \| - \| - \| 0.709873 \| 0.000023 \| \| 19 \| Tq-Bs-18 \| Tarq_Comp_Mon \| arch \| - \| - \| pig_enamel \| - \| - \| - \| - \| - \| - \| 0.709007 \| 0.000026 \| \| 20 \| Tq-Bs-19 \| Tarq_Comp_Mon \| arch \| - \| - \| pig_enamel \| - \| - \| - \| - \| - \| - \| 0.710216 \| 0.000023 \| \| 21 \| Tq-Bs-20 \| Tarq_Comp_Mon \| arch \| - \| - \| pig_enamel \| - \| - \| - \| - \| - \| - \| 0.709151 \| 0.000022 \| \| 22 \| Tq-Bs-21 \| Ara PC \| arch \| - \| - \| hare_enamel \| - \| - \| - \| - \| - \| - \| 0.709432 \| 0.000022 \| \| 23 \| Tomb_234 \| Tarq_Villa_Br \| arch \| - \| YC \| homo_LM2 \| - \| - \| - \| - \| - \| - \| 0.709527 \| 0.000020 \| \| 24 \| Tomb_136 \| Tarq_Villa_Br \| arch \| - \| YC \| homo_LPB \| - \| - \| - \| - \| - \| - \| 0.709176 \| 0.000013 \| \| 25 \| Tomb_202 \| Tarq_Villa_Br \| arch \| - \| OC \| ­ homo_M1/M2(?) \| - \| - \| - \| - \| - \| - \| 0.709358 \| 0.000010 \| \| 26 \| Tomb_205 \| Tarq_Villa_Br \| arch \| - \| YC \| homo_RPB \| - \| - \| - \| - \| - \| - \| 0.709072 \| 0.000011 \| \| 27 \| Tomb_26 \| Tarq_Villa_Br \| arch \| - \| OC \| homo_LPB \| - \| - \| - \| - \| - \| - \| 0.709290 \| 0.000013 \| \| 28 \| Tomb_64 \| Tarq_Villa_Br \| arch \| - \| OC \| homo_C \| - \| - \| - \| - \| - \| - \| 0.709301 \| 0.000007 \| \| 29 \| Tomb_159 \| Tarq_Villa_Br \| arch \| - \| YC \| homo_LPB \| - \| - \| - \| - \| - \| - \| 0.709267 \| 000009 \| \| 30 \| Tomb_111 \| Tarq_Villa_Br \| arch \| - \| OC \| homo_M2(?) \| - \| - \| - \| - \| - \| - \| 0.709173 \| 0.000008 \| \| 31 \| Tomb_207 \| Tarq_Villa_Br \| arch \| - \| YC \| homo_LPB \| - \| - \| - \| - \| - \| - \| 0.709342 \| 0.000008 \| \| 32 \| Tomb_147 \| Tarq_Villa_Br \| arch \| - \| YC \| homo_RPB \| - \| - \| - \| - \| - \| - \| 0.709236 \| 0.000007 \| |
| --- | --- | --- | --- | --- | --- | --- | --- | --- | --- | --- | --- | --- | --- | --- | --- | --- | --- | --- | --- | --- | --- | --- | --- | --- | --- | --- | --- | --- | --- | --- | --- | --- | --- | --- | --- | --- | --- | --- | --- | --- | --- | --- | --- | --- | --- | --- | --- | --- | --- | --- | --- | --- | --- | --- | --- | --- | --- | --- | --- | --- | --- | --- | --- | --- | --- | --- | --- | --- | --- | --- | --- | --- | --- | --- | --- | --- | --- | --- | --- | --- | --- | --- | --- | --- | --- | --- | --- | --- | --- | --- | --- | --- | --- | --- | --- | --- | --- | --- | --- | --- | --- | --- | --- | --- | --- | --- | --- | --- | --- | --- | --- | --- | --- | --- | --- | --- | --- | --- | --- | --- | --- | --- | --- | --- | --- | --- | --- | --- | --- | --- | --- | --- | --- | --- | --- | --- | --- | --- | --- | --- | --- | --- | --- | --- | --- | --- | --- | --- | --- | --- | --- | --- | --- | --- | --- | --- | --- | --- | --- | --- | --- | --- | --- | --- | --- | --- | --- | --- | --- | --- | --- | --- | --- | --- | --- | --- | --- | --- | --- | --- | --- | --- | --- | --- | --- | --- | --- | --- | --- | --- | --- | --- | --- | --- | --- | --- | --- | --- | --- | --- | --- | --- | --- | --- | --- | --- | --- | --- | --- | --- | --- | --- | --- | --- | --- | --- | --- | --- | --- | --- | --- | --- | --- | --- | --- | --- | --- | --- | --- | --- | --- | --- | --- | --- | --- | --- | --- | --- | --- | --- | --- | --- | --- | --- | --- | --- | --- | --- | --- | --- | --- | --- | --- | --- | --- | --- | --- | --- | --- | --- | --- | --- | --- | --- | --- | --- | --- | --- | --- | --- | --- | --- | --- | --- | --- | --- | --- | --- | --- | --- | --- | --- | --- | --- | --- | --- | --- | --- | --- | --- | --- | --- | --- | --- | --- | --- | --- | --- | --- | --- | --- | --- | --- | --- | --- | --- | --- | --- | --- | --- | --- | --- | --- | --- | --- | --- | --- | --- | --- | --- | --- | --- | --- | --- | --- | --- | --- | --- | --- | --- | --- | --- | --- | --- | --- | --- | --- | --- | --- | --- | --- | --- | --- | --- | --- | --- | --- | --- | --- | --- | --- | --- | --- | --- | --- | --- | --- | --- | --- | --- | --- | --- | --- | --- | --- | --- | --- | --- | --- | --- | --- | --- | --- | --- | --- | --- | --- | --- | --- | --- | --- | --- | --- | --- | --- | --- | --- | --- | --- | --- | --- | --- | --- | --- | --- | --- | --- | --- | --- | --- | --- | --- | --- | --- | --- | --- | --- | --- | --- | --- | --- | --- | --- | --- | --- | --- | --- | --- | --- | --- | --- | --- | --- | --- | --- | --- | --- | --- | --- | --- | --- | --- | --- | --- | --- | --- | --- | --- | --- | --- | --- | --- | --- | --- | --- | --- | --- | --- | --- | --- | --- | --- | --- | --- | --- | --- | --- | --- | --- | --- | --- | --- | --- | --- | --- | --- | --- | --- | --- | --- | --- | --- | --- | --- | --- | --- | --- | --- | --- | --- | --- | --- | --- | --- | --- | --- | --- | --- | --- | --- | --- | --- | --- | --- | --- |
|  |

Table S4 – Summary of individual information. including uniparental haplogroups and contamination estimates. Endo. = Endogenous. Hg = Haplogroup. MD = Molecular Damage, Mito. = Mitochondrial.

| **Individual** | **Genetic Sex** | **Endo. DNA** | **Genomic**  **Coverage (X)** | **Final no. aligned reads** | **Mito. Hg** | **Mito. contam. + MD** | **Mito. contam. - MD** | **Y Hg** | **X-chr contam.** |
| --- | --- | --- | --- | --- | --- | --- | --- | --- | --- |
| Individual 8 | F | 58% | 1.36 | 67,441,527 | HV+16311 | 0.0098 | 0.0088 | - | - |
| Individual 10 | M | 18% | 0.86 | 43,720,002 | U5b2b3 | 0.0102 | 0.0114 | J-M12 (J2b) | 0.028 |
| Individual 11 | F | 19% | 0.96 | 49,833,896 | H3 | 0.0123 | 0.0123 | - | - |
| Individual 12 | F | 3% | - | - | - | - | - | - | - |
| Individual 14 | F | 51% | 1.85 | 88,304,305 | T2 | 0.008 | 0.0098 | - | - |
| Individual 19 | M | 41% | 1.7 | 83,252,084 | T2b | 0.0073 | 0.0095 | J-M241 (J2b2a) | 0.011 |

Table S5 – Accepted single source models for Individual 11. Models are considered accepted if p-value >0.05. marginally accepted if >0.01 (*).

| **Population** | **Population Verbose** | **p-value for model** |
| --- | --- | --- |
| Sweden_IA | Sweden Iron Age | 0.2256 |
| Norway_IA | Norway Iron Age | 0.0975 |
| Denmark_IA | Denmark Iron Age | 0.0757 |
| Scotland_IA | Scotland Iron Age | 0.0499 |
| France_IA_North | North France Iron Age | 0.0212* |

Table S6 – Pigmentation profile results for imputed Iron Age Italians using the h-Irisplex-S system. **Brown not over the stated threshold of 0.7 but has the highest probability. with intermediate as second. ~~The category of Blond/Light Brown is considered to refer to the same colour.~~

| **Context** | **Sample** | **Eye** | **Hair** | **Skin** |
| --- | --- | --- | --- | --- |
| Adriatic coast  Final Bronze Age | R1 | Blue | Blond/Light Brown | Pale-Intermediate |
| Etruscan | R1015 | Blue | Brown | Intermediate |
| Etruscan | **Individual 14** | Brown | Dark Brown | Intermediate-Dark |
| Etruscan | **Individual 11** | Brown** | Lighter Brown | Intermediate |
| Etruscan | **Individual 10** | Blue | Brown/Dark Brown | Intermediate |
| Etruscan | **Individual 19** | Blue | Lighter Brown | Intermediate |
| Etruscan | **Individual 8** | Blue | Lighter Brown | Pale-Intermediate |
| Latin | R435 | Brown | Dark Brown | Intermediate-Dark |
| Latin | R437 | Brown | Dark Brown | Intermediate-Dark |
| Etruscan | R473 | Brown | Lighter Brown | Intermediate |
| Latin | R1016 | Brown | Dark Brown | Intermediate-Dark |
| Latin | R1021 | Brown | Lighter Brown | Intermediate |
| Etruscan | R474 | Brown | Blond/Light Brown | Intermediate |
| Etruscan | R475 | Brown | Lighter Brown (with red tones) | Pale-Intermediate |
| Latin | R850 | Brown | Dark Brown/Black | Intermediate |
| Latin | R851 | Brown | Dark Brown | Dark |

#Table S7 Here

#Table S8 Here

#Table S9 Here

#Table S10 Here

#Table S11 Here

#Table S12 Here

# **Supplementary code**

OxCal CQL2 model for the model of Table S1A

Plot()

{

Curve("Atmospheric","intcal20.14c");

Curve("Marine","marine20.14c");

Delta_R("Med",-150,60);

Sequence("Stratigraphy under 1524")

{

Boundary("Start of stratigraphy under 1524");

Phase("Burials under 1609")

{

Mix_Curves("50pc marine diet","Atmospheric","Med",50,10);

R_Date("Individual 11 UBA-43006",2747,36) & Date(U(-900,-750,1));

Mix_Curves("25pc marine diet","Atmospheric","Med",25,10);

R_Date("Individual 12 UBA-43007",2792,40) & Date(U(-900,-750,1));

};

Boundary("Layer 1524");

Curve("Atmospheric","intcal20.14c");

R_Date("Charcoal UBA-42280",2602,36);

Boundary("End of stratigraphy under 1524");

};

Phase("Burials elsewhere")

{

Mix_Curves("25pc marine diet","Atmospheric","Med",25,10);

R_Date("Individual 14 UBA-43008",2828,28);

R_Date("Individual 10 UBA-43005",2618,32);

Mix_Curves("70pc marine diet","Atmospheric","Med",70,10);

R_Date("Individual 19 UBA-43009",2709,24);

Mix_Curves("65pc marine diet","Atmospheric","Med",65,10);

R_Date("Individual 8 UBA-43004",2620,35);

// LTL dates have no d13C so marine component is unknown

Mix_Curves("Unknown marine diet","Atmospheric","Med",50,50);

R_Date("Individual 9 LTL-20257A",2387,45);

R_Date("Individual 15 LTL-20258A",2419,45);

Sequence("Individuals 16 and 18")

{

Boundary("Start of Ind. 18-16 sequence");

Curve("Atmospheric","intcal20.14c");

After(R_Date("Individual 18 LTL-20260A",2892,45));

Boundary("3310 / 3311 boundary");

Mix_Curves("Unknown marine diet","Atmospheric","Med",50,50);

R_Date("Individual 16 LTL-20259A",2578,45);

Boundary("End of Ind. 18-16 sequence");

};

};

};

# **References**

^1^ Stoddart, S. *Power and Place in Etruria. The spatial dynamics of a Mediterranean civilisation. 1200-500 B.C*. (Cambridge University Press, 2020).

^2^ Haynes, S. *Etruscan Civilisation. A Cultural History*. (British Museum Press, 2000).

^3^ Leighton, R. *Tarquinia. An Etruscan City*. (Duckworth, 2004).

^4^ Bonghi Jovino, M. The Tarquinia Project: A Summary of 25 Years of Excavation. *American Journal of Archaeology* **114**, 161-180 (2010).

^5^Marzullo, M. *Tarquinia. L’abitato e le sue mura. Indagini di topografia storica*. (Ledizioni, 2018).

^6^Bagnasco Gianni, G. in *Aristonothos 14* (Milano University Press, 2018).

^7^ Marzullo, M. *Spazi Sepolti e dimensioni dipinte nelle tombe etrusche di Tarquinia*. (Ledizioni, 2017).

^8^ Bagnasco Gianni, G., Marzullo, M. & Piazzi, C. in *Making Cities Economies of Production and Urbanisation in Mediterranean Europe 1000–500 BCE* (eds Margarita Gleba, Beatrice Marin Aguilera, & Bela Dimova) 177-193 (McDonald, 2021).

^9^Quilici Gigli, S. *Tuscana*. Forma Italiae. 2 Regio 7; (De Luca, 1970).

^10^Barker, G. & Rasmussen, T. The archaeology of an etruscan polis: a preliminary report on the Tuscania project. 1986 and 1987 seasons. *Papers of the British School at Rome* **56**, 25-42 (1988).

^11^Perego, L. G. *Il territorio tarquiniese : ricerche di topografia storica*. Il filarete / Università degli studi di Milano, Facoltà di lettere e filosofia, Sezione di storia dell'arte 229 (LED, 2005).

^12^Bagnasco Gianni, G., Facchetti, F. M., Cattaneo, C., Maderna, E. & Ricciardi, V. in *Una favola breve. Archeologia e antropologia per la storia dell’infanzia* (ed C Lambrugo) 211-224 (All'Insegna del Giglio, 2019).

^13^Bonghi Jovino, M. & Chiesa, F. Offerte dal regno vegetale e dal regno animale nelle manifestazioni del sacro. Atti dell'incontro di studio Milano 26-27 giugno 2003 in *Università degli Studi di Milano. Tarchna. Supplemento 1* (L'Erma di Bretschneider, 2005).

^14^Bagnasco Gianni, G., Cattaneo, C., Marzullo, M., Mazzarelli, D. & Ricciardi, V. in *BIRTH. Archeologia dell'infanzia nell'Italia preromana* (ed E Govi) 333-359 (Bologna University Press, 2021).

^15^Funiciello, R., De Rita, D. & Sposato, A. *Note illustrative della Carta Geologica d’Italia alla scala 1:50.000, Foglio 354 Tarquinia*. (ISPRA Servizio Geologico d’Italia, 2012).

^16^Lugli, F. *et al.* A strontium isoscape of Italy for provenance studies. *Chemical Geology* **587**, 120624, doi:https://doi.org/10.1016/j.chemgeo.2021.120624 (2022).

^17^Cattuto, C., Gregori, L., Milano, M. & Rapicetta, S. Condizioni geomorfologiche e stabilita’ dell’acropoli di Tarquinia Vecchia. *Il Quaternario-Italian Journal of Quaternary Sciences* **19**, 227-232 (2006).

^18^Chiocchini, U. & Castaldi, F. Caratteri sedimentologici e composizionali delle ghiaie del sintema di Poggio Martino, Bacino Plio-Pleistocenico di Tarquinia, Italia centrale. *Italian .Journal of Geosciences Bollettino.Societa Geologica Italiana* **128**, 695-713 (2009).

^19^De Rita, D., Fabbri, M. & Cimarelli, C. Evoluzione pleistocenica del margine tirrenico dell’Italia centrale tra eustatismo, vulcanismo e tettonica. *Il Quaternario-Italian Journal of Quaternary Sciences* **17**, 523-536 (2004).

^20^Napoli, R., Paolanti, M., Rivieccio, R. & Di Ferdinando, S. *Carta dei suoli del Lazio Scala 1:250000. Programma Interregionale Agricoltura Qualità - Misura 5 - Realizzazione della Carta Pedologica Nazionale 1:250 000*. (L’Agenzia Regionale per lo Sviluppo e l’Innovazione dell’Agricoltura del Lazio (ARSIAL), 2019).

^21^Bagnasco Gianni, G. Tarquinii in *Oxford Classical Dictionary, digital ed. Oxford University Press.* (ed Tim Whitmarsh) doi: 10.1093/acrefore/9780199381135.9780199381013.9780199386226 (Oxford University Press, 2021).

^22^Bagnasco Gianni, G. Architectural Choices in Etruscan Sacred Areas: Tarquinia in Its Mediterranean Setting in *Architecture in Ancient Central Italy: Connections in Etruscan and Early Roman Building British School at Rome Studies* (ed in C. Potts (ed.)) 148-173 (Cambridge University Press, 2022).

^23^Bagnasco Gianni, G. Quale Hercle nella Roma di Tarquinio il Superbo?, in The Age of Tarquinius Superbus. Central Italy in the Late 6th Century in *Proceedings of the Conference The Age of Tarquinius Superbus, A Paradigm Shift? Rome, 7-9 November 2013* (eds Patricia S Lulof & Christopher J Smith) 159-167 (Peeters, 2017).

^24^G. Bagnasco Gianni (ed.), Un’áncora sul Pianoro della Civita di Tarquinia (Tarquinia, 12 Ottobre 2013), Aristonothos 10, 2015.

^25^ Bagnasco Gianni, G. in *Cên zic ziχuχe. Per Maristella Pandolfini* (ed Enrico Benelli) 21-26 (Fabrizio Serra Editore, 2014).

^26^ Bonghi Jovino, M. in *Italo - Tusco - Romana: Festschrift für Luciana Aigner-Foresti zum 70. Geburtstag am 30. Juli 2006* (eds P. Amann-M. & H. Taeuber (a cura di) Pedrazzi) 39-45 (Holzhausen, 2006).

^27^ Bonghi Jovino, M. in *Material aspects of Etruscan Religion. Proceedings of the International Colloquium (Leiden, May 29th and 30th, 2008)* *Papers on Mediterranean Archaeology, Supplement 16* (ed L.B van der Meer) 5-16 (Peeters, 2010).

^28^ Bagnasco Gianni, G., Marzullo, M., Piazzi, C. & Garzulino, A. Richerche nell'area urbana di Tarquinia. *Annali della Fondazione per il Museo “C. Faina”* **25**, 281-341 (2018).

^29^Rodella, L. *et al.* in *14th Congress of the Italian Anthropological Association. Chieti* (2022).

^30^ Kanz, F. & Grossschmidt, K. in *Roman Amphitheatres and Spectacula, a 21st-century Perspective: Papers from an International Conference Held at Chester, 16th-18th February, 2007* (ed Tony Wilmott) 211-220 (British Archaeological Reports Limited, 2009).

^31^ Gilmour, R. J. *et al.* Gendered differences in accidental trauma to upper and lower limb bones at Aquincum, Roman Hungary. *International journal of paleopathology* **11**, 75-91 (2015).

^32^ Klales, A. R., Ousley, S. D. & Vollner, J. M. A revised method of sexing the human innominate using Phenice’s nonmetric traits and statistical methods. *American Journal of Physical Anthropology* **149**, 104–114. https://doi.org/110.1002/ajpa.22102 (2012).

^33^ Phenice, T. W. A newly developed visual method of sexing the os pubis. *American Journal of Physical Anthropology* **30**, 297–301. https://doi.org/https://doi.org/210.1002/ajpa.1330300214 (1969).

^34^ Walker, P. L. Sexing skulls using discriminant function analysis of visually assessed traits. *American Journal of Physical Anthropology* **136**, 39–50. https://doi.org/https://doi.org/10.1002/ajpa.20776 (2008).

^35^ Walker, P. L. Greater sciatic notch morphology: Sex, age, and population differences. . *American Journal of Physical Anthropology* **127**, 385–391. https://doi.org/https://doi.org/310.1002/ajpa.10422 (2005).

^36^ Purkait, R. Sex determination from femoral head measurements: a new approach. *Legal Medicine* **5**, S347-S350, doi:https://doi.org/10.1016/S1344-6223(02)00169-4 (2003).

^37^ Spradley, M. K. & Jantz, R. L. Sex estimation in forensic anthropology: Skull versus postcranial elements. *Journal of Forensic Sciences* **56,** 289–296. https://doi.org/210.1111/j.1556-4029.2010.01635.x (2011).

^38^ Brooks, S. & Suchey, J. M. Skeletal age determination based on the os pubis: A comparison of the Acsádi-Nemeskéri and Suchey-Brooks methods. *Human Evolution* **5**, 227–238. https://doi.org/210.1007/BF02437238 (1990).

^39^ Buckberry, J. L. & Chamberlain, A. T. Age estimation from the auricular surface of the ilium: A revised method. *American Journal of Physical Anthropology* **119**, 231–239 (2002).

^40^ Lovejoy, C. O., Meindl, R. S., Pryzbeck, T. R. & Mensforth, R. P. Chronological metamorphosis of the auricular surface of the ilium: A new method for the determination of adult skeletal age at death. *American Journal of Physical Anthropology* **68**, 15–28. https://doi.org/10.1002/ajpa.1330680103 (1985).

^41^ Rougé-Maillart, C. *et al.* Development of a method to estimate skeletal age at death in adults using the acetabulum and the auricular surface on a Portuguese population. *Forensic Science International* **188**, 91–95. https://doi.org/10.1016/j.forsciint.2009.1003.1019 (2009).

^42^ Işcan, M. Y. & Loth, S. R. Determination of age from the sternal rib in white females: a test of the phase method. *Journal of Forensic Sciences* **31**, 990-999 (1986).

^43^ Kvaal, S. I., Kolltveit, K. M., Solheim, T. & Thomsen, I. Age estimation of adults from dental radiographs. *Forensic Science International* **74**, 175–185 (1995).

^44^ Hefner, J. T. Cranial Nonmetric Variation and Estimating Ancestry*. *Journal Forensic Science* **54**, 985–995. https://doi.org/910.1111/j.1556-4029.2009.01118.x (2009).

^45^ Hefner, J. T. & Ousley, S. D. Statistical classification methods for estimating ancestry using morphoscopic traits. *Journal of Forensic Sciences* **59**, 883–890. https://doi.org/810.1111/1556-4029.12421 (2014).

^46^ Aufderheide, A. C. & Rodríguez-Martín, C. The Cambridge Encyclopedia of Human Paleopathology (Cambridge University Press, Cambridge, 1998).

^47^ Biehler-Gomez, L. & Cattaneo, C. *Interpreting bone lesions and pathology for forensic practice*. (Academic Press, 2020).

^48^ Buikstra, J. E. *Ortner's identification of pathological conditions in human skeletal remains*. (Elsevier, 2019).

^49^ Ortner, D. J. *Identification of Pathological Conditions in Human Skeletal Remains*. Third edn, (Smithsonian Institution Press, 2003).

^50^ Christensen, A. M., Passalacqua, N. V. & Bartelink, E. J. *Forensic anthropology: current methods and practice*. (Academic Press, 2014).

^51^ Kimmerle, E. H. & Baraybar, J. P. *Skeletal trauma: identification of injuries resulting from human rights abuse and armed conflict*. (Taylor & Francis, 2008).

^52^ Wedel, V. L. & Galloway, A. (eds) *Broken Bones: Anthropological Analysis of Blunt Force Trauma*. (Charles C Thomas Publisher, 2014).

^53^Shriver-Rice, M. & Schmidt, F. Environmental and Archaeobotanical Studies in Etruscan Archaeology: An Epistemological Overview and Future Considerations of Human–Plant Relationships. *Etruscan and Italic Studies* **25**, 113-147, doi:doi:10.1515/etst-2022-0001 (2022).

^54^ Reimer, P. J. *et al.* Selection and Treatment of Data for Radiocarbon Calibration: An Update to the International Calibration (IntCal) Criteria. *Radiocarbon* **55**, 1923-1945, doi:10.2458/azu_js_rc.55.16955 (2013).

^55^Govan, E. & Parnell, A. simmr: A Stable Isotope Mixing Model. R package version 0.5.1.212. https://CRAN.R-project.org/package=simmr. (2023).

^56^ McLaughlin, R. *et al.* in *Temple People: Bioarchaeology, Resilience and Culture in Prehistoric Malta. Volume 3 of Fragility and Sustainability – Studies in Early Malta, the ERC-funded FRAGSUS Project* (eds S. Stoddart *et al.*) 295-302 (McDonald Institute for Archaeological Research, 2022).

^57^ Reimer, P. J. *et al.* The IntCal20 Northern Hemisphere Radiocarbon Age Calibration Curve (0–55 cal kBP). *Radiocarbon* **62**, 725-757, doi:10.1017/RDC.2020.41 (2020).

^58^ Heaton, T. J. *et al.* Marine20—The Marine Radiocarbon Age Calibration Curve (0–55,000 cal BP). *Radiocarbon* **62**, 779-820, doi:10.1017/RDC.2020.68 (2020).

^59^ Bronk Ramsey, C. Bayesian analysis of radiocarbon dates. *Radiocarbon* **51**, 337–360 (2009).

^60^ Balasse, M. Reconstructing dietary and environmental history from enamel isotopic analysis: Time resolution of intra-tooth sequential sampling. *International Journal of Osteoarchaeology* **12**, 155-165 (2002).

^61^ Coplen, T. B. Reporting of stable hydrogen, carbon, and oxygen isotopic abundances. *Geothermics* **24**, 707-712, doi:https://doi.org/10.1016/0375-6505(95)00024-0 (1995).

^62^ Hoefs, J. *Stable Isotope Geochemistry*. 4th edition edn (Springer-Verlag, 1997).

^63^ Esposito, C. *et al.* Intense community dynamics in the pre-Roman frontier site of Fermo (ninth–fifth century BCE, Marche, central Italy) inferred from isotopic data *Scientific Reports* **13**, 3632, doi:10.1038/s41598-023-29466-3 (2023).

^64^ Veselka, B. *et al.* Strontium isotope ratios related to childhood mobility: Revisiting sampling strategies of the calcined human pars petrosa ossis temporalis. *Rapid Communications in Mass Spectrometry* **35 (7)**, e9038. doi: 9010.1002/rcm.9038. PMID: 33370492 (2021).

^65^ Snoeck C, Lee-Thorp J, Schulting R, De Jong J, Debouge W, Mattielli N. Calcined bone provides a reliable substrate for strontium isotope ratios as shown by an enrichment experiment. *Rap Commun Mass Spectrom*. 2015;29:107-114. DOI: 10.1002/recm.7078

^66^ Müller, W., Fricke, H., Halliday, A. N., McCulloch, M. T. & Wartho, J. A. Origin and migration of the Alpine Iceman. *Science* **302**, 862–866 (2003).

^67^ Scorrer, J. *et al.* Diversity aboard a Tudor warship: investigating the origins of the Mary Rose crew using multi-isotope analysis. *Royal Society Open Science* **8**, 202106. (2021).

^68^ Font, L., Nowell, G. M., Graham Pearson, D., Ottley, C. J. & Willis, S. G. Sr isotope analysis of bird feathers by TIMS: a tool to trace bird migration paths and breeding sites. *Journal of Analytical Atomic Spectrometry* **22**, 513–522. doi:510.1039/b616328a (2007).

^69^ Müller, W. & Anczkiewicz, R. Accuracy of laser-ablation (LA)-MC-ICPMS Sr isotope analysis of (bio)apatite – a problem reassessed. *Journal of Analytical Atomic Spectrometry* **31**, 259-269 (2016).

^70^ McArthur, J. M., Howarth, R. J. & Bailey, T. R. Strontium Isotope Stratigraphy: LOWESS Version 3: Best Fit to the Marine Sr‐Isotope Curve for 0–509 Ma and Accompanying Look‐up Table for Deriving Numerical Age. *The Journal of Geology* **109**, 155-170, doi:10.1086/319243 (2001).

^71^ Nier, A. O. The isotopic constitution of strontium, barium, bismuth, thallium and mercury. . *Physical Review* **54**, 275–278. doi:210.1103/ PhysRev.1154.1275 (1938).

^72^ Avanzinelli, R. *et al.* High precision Sr, Nd, and Pb isotopic analyses using the new generation thermal ionisation mass spectrometer thermofinnigan triton-Ti®. *Periodico di Mineralogia* **74**, 147–166 (2005).

^73^ Romaniello, S. J. *et al.* Fully automated chromatographic purification of Sr and Ca for isotopic analysis. *Journal of Analytical Atomic Spectrometry* **30**, 1906-1912, doi:10.1039/C5JA00205B (2015).

^74^ Yang, D. Y., Eng, B., Waye, J. S., Dudar, J. C. & Saunders, S. R. Improved DNA extraction from ancient bones using silica-based spin columns. *Am. J. Phys. Anthropol.* **105,** 539–543, [doi:10.1002/(SICI)1096-8644(199804)105:4%3C539::AID-AJPA10%3E3.0.CO;2-1](https://doi.org/10.1002/(SICI)1096-8644(199804)105:4%3C539::AID-AJPA10%3E3.0.CO;2-1) (1998)

^75^ MacHugh, D. E., Edwards, C. J., Bailey, J. F., Bancroft, D. R. & Bradley, D. G. The Extraction and Analysis of Ancient DNA from Bones and Teeth: a Survey of Current Methodologies. *Anc. Biomol.* **3**, 81 (2000)

^76^ Boessenkool, S. *et al.* Combining bleach and mild predigestion improves ancient DNA recovery from bones. *Mol. Ecol. Resour.* **17**, 742–751, doi: 10.1111/1755-0998.12623 (2017)

^77^ Meyer, M. & Kircher, M. Illumina Sequencing Library Preparation for Highly Multiplexed Target Capture and Sequencing. *Cold Spring Harbor protocols* **2010**, pdb.prot5448, doi:10.1101/pdb.prot5448 (2010).

^78^ Gamba, C. *et al.* Genome flux and stasis in a five millennium transect of European prehistory. *Nat. Commun.* **5**, 5257, doi:10.1038/ncomms6257 (2014).

^79^ Jónsson, H., Ginolhac, A., Schubert, M., Johnson, P.L.F., and Orlando, L. (2013). mapDamage2.0: fast approximate Bayesian estimates of ancient DNA damage parameters. Bioinformatics 29, 1682–1684

^80^ Schubert, M., Lindgreen, S. & Orlando, L. AdapterRemoval v2: rapid adapter trimming, identification, and read merging. *BMC Research Notes* **9**, 88, doi:10.1186/s13104-016-1900-2 (2016).

^81^ Li, H. & Durbin, R. Fast and accurate short read alignment with Burrows–Wheeler transform. *Bioinformatics* **25**, 1754-1760, doi:10.1093/bioinformatics/btp324 (2009).

^82^ Li, H. *et al.* The Sequence Alignment/Map format and SAMtools. *Bioinformatics* **25**, 2078-2079, doi:10.1093/bioinformatics/btp352 (2009).

^83^ [(2019). Picard toolkit. Broad Institute, GitHub Repository.](http://paperpile.com/b/4MPIdA/Affhs)

^84^ McKenna, A. *et al.* The Genome Analysis Toolkit: a MapReduce framework for analyzing next-generation DNA sequencing data. *Genome Res* **20**, 1297-1303, doi:10.1101/gr.107524.110 (2010).

^85^ Okonechnikov, K., Conesa, A. & García-Alcalde, F. Qualimap 2: advanced multi-sample quality control for high-throughput sequencing data. *Bioinformatics* **32**, 292–294 (2016).

^86^ Skoglund, P., Storå, J., Götherström, A. & Jakobsson, M. Accurate sex identification of ancient human remains using DNA shotgun sequencing. *Journal of Archaeological Science* **40**, 4477-4482, doi:https://doi.org/10.1016/j.jas.2013.07.004 (2013).

^87^ Danecek, P. *et al.* Twelve years of SAMtools and BCFtools. *Gigascience* **10**, doi:10.1093/gigascience/giab008 (2021).

^88^ Weissensteiner, H. *et al.* HaploGrep 2: mitochondrial haplogroup classification in the era of high-throughput sequencing. *Nucleic Acids Res* **44**, W58-63, doi:10.1093/nar/gkw233 (2016).

^89^ van Oven, M. & Kayser, M. Updated comprehensive phylogenetic tree of global human mitochondrial DNA variation. *Human Mutation* **30**, E386-E394, doi:https://doi.org/10.1002/humu.20921 (2009).

^90^ International Society of Genetic Genealogy. Y-DNA Haplogroup Tree 2019, Version: 15.73, Date: 11 July 2020.

^91^ Korneliussen, T. S., Albrechtsen, A. & Nielsen, R. ANGSD: Analysis of Next Generation Sequencing Data. *BMC Bioinformatics* **15**, 356, doi:10.1186/s12859-014-0356-4 (2014).

^92^ Rasmussen, M. *et al.* An Aboriginal Australian genome reveals separate human dispersals into Asia. *Science* **334**, 94–98, doi:10.1126/science.1211177 (2011).

^93^ Mathieson, I. *et al.* Genome-wide patterns of selection in 230 ancient Eurasians. *Nature* **528**, 499–503, doi:10.1038/nature16152 (2015).

^94^ Martiniano, R. *et al.* The population genomics of archaeological transition in west Iberia: Investigation of ancient substructure using imputation and haplotype-based methods. *PLoS Genet.* **13**, e1006852, doi:10.1371/journal.pgen.1006852 (2017).

^95^ Allentoft, M. E. *et al.* Population genomics of Bronze Age Eurasia. *Nature* **522**, 167–172, doi:10.1038/nature14507 (2015).

^96^ Antonio, M. L. *et al.* Ancient Rome: A genetic crossroads of Europe and the Mediterranean. *Science* **366**, 708-714, doi:10.1126/science.aay 6826 (2019).

^97^ Brace, S. *et al.* Ancient genomes indicate population replacement in Early Neolithic Britain. *Nat Ecol Evol* **3**, 765–771, doi:10.1038/s41559-019-0871-9 (2019).

^98^ Broushaki, F. *et al.* Early Neolithic genomes from the eastern Fertile Crescent. *Science* **353**, 499–503, doi:10.1126/science.aaf7943 (2016).

^99^ Brunel, S. *et al.* Ancient genomes from present-day France unveil 7,000 years of its demographic history. *Proc. Natl. Acad. Sci. U. S. A.* **117**, 12791–12798, doi:10.1073/pnas.1918034117 (2020).

^100^ Cassidy, L. M. *et al.* Neolithic and Bronze Age migration to Ireland and establishment of the insular Atlantic genome. *Proc. Natl. Acad. Sci. U. S. A.* **113**, 368–373, doi:10.1073/pnas.1518445113 (2016)

^101^ Cassidy, L. M. *et al.* A dynastic elite in monumental Neolithic society. *Nature* **582**, 384–388, doi:10.1038/s41586-020-2378-6 (2020).

^102^ Fregel, R. *et al.* Ancient genomes from North Africa evidence prehistoric migrations to the Maghreb from both the Levant and Europe. *Proc. Natl. Acad. Sci. U. S. A.* **115**, 6774–6779, doi:10.1073/pnas.1800851115 (2018).

^103^ Fu, Q. *et al.* Genome sequence of a 45,000-year-old modern human from western Siberia. *Nature* **514**, 445–449, doi:10.1038/nature13810 (2014).

^104^ Fu, Q. *et al.* The genetic history of Ice Age Europe. *Nature* **534**, 200–205, doi:10.1038/nature17993 (2016).

^105^ Gallego-Llorente, M. *et al.* The genetics of an early Neolithic pastoralist from the Zagros, Iran. *Sci. Rep.* **6**, 31326, doi:10.1038/srep31326 (2016)

^106^ Gallego Llorente, M. *et al.* Ancient Ethiopian genome reveals extensive Eurasian admixture in Eastern Africa. *Science* **350**, 820–822, doi:10.1126/science.aad2879 (2015).

^107^ Haak, W. *et al.* Massive migration from the steppe was a source for Indo-European languages in Europe. *Nature* **522**, 207–211, doi:10.1038/nature14317 (2015).

^108^ Hofmanová, Z. *et al.* Early farmers from across Europe directly descended from Neolithic Aegeans. *Proceedings of the National Academy of Sciences* **113**, 6886–6891, doi:10.1073/pnas.1523951113 (2016).

^109^ Jones, E. R. *et al.* Upper Palaeolithic genomes reveal deep roots of modern Eurasians. *Nat. Commun.* **6**, 8912, doi:10.1038/ncomms9912 (2015).

^110^ Kılınç, G. M. *et al.* The Demographic Development of the First Farmers in Anatolia. *Curr. Biol.* **26**, 2659–2666, doi:10.1016/j.cub.2016.07.057 (2016).

^111^ Lazaridis, I. *et al.* Ancient human genomes suggest three ancestral populations for present-day Europeans. *Nature* **513**, 409–413, doi:10.1038/nature13673 (2014).

^112^ Lazaridis, I. *et al.* Genomic insights into the origin of farming in the ancient Near East. *Nature* **536**, 419–424, doi:10.1038/nature19310 (2016).

^113^ Marcus, J. H. *et al.* Genetic history from the Middle Neolithic to present on the Mediterranean island of Sardinia. *Nat. Commun.* **11**, 939, doi:10.1038/s41467-020-14523-6 (2020).

^114^ Margaryan, A. *et al.* Population genomics of the Viking world. *Nature* **585**, 390–396, doi:10.1038/s41586-020-2688-8 (2020).

^115^ Martiniano, R. *et al.* Genomic signals of migration and continuity in Britain before the Anglo-Saxons. *Nat. Commun.* **7**, 10326, doi:10.1038/ncomms10326 (2016).

^116^ Mathieson, I. *et al.* The genomic history of southeastern Europe. *Nature* **555**, 197–203, doi:10.1038/nature25778 (2018).

^117^ Olalde, I. *et al.* The Beaker phenomenon and the genomic transformation of northwest Europe. *Nature* **555**, 190–196, doi:10.1038/nature25738 (2018).

^118^ Olalde, I. *et al.* The genomic history of the Iberian Peninsula over the past 8000 years. *Science* **363**, 1230–1234, doi:10.1126/science.aav4040 (2019).

^119^ Posth, C. *et al.* The origin and legacy of the Etruscans through a 2000-year archeogenomic time transect. *Science Advances* **7**, eabi7673, doi:10.1126/sciadv.abi7673 (2021).

^120^ Raghavan, M. *et al.* Upper Palaeolithic Siberian genome reveals dual ancestry of Native Americans. *Nature* **505**, 87–91, doi:10.1038/nature12736 (2014).

^121^ Rivollat, M. *et al.* Ancient genome-wide DNA from France highlights the complexity of interactions between Mesolithic hunter-gatherers and Neolithic farmers. *Sci Adv* **6**, eaaz5344, doi:10.1126/sciadv.aaz5344 (2020).

^122^ Saag, L. *et al.* The Arrival of Siberian Ancestry Connecting the Eastern Baltic to Uralic Speakers further East. *Curr. Biol.* **29**, 1701–1711.e16 , doi:10.1016/j.cub.2019.04.026 (2019).

^123^ Saupe, T. *et al.* Ancient genomes reveal structural shifts after the arrival of Steppe-related ancestry in the Italian Peninsula. *Curr. Biol.* **31**, 2576–2591.e12, doi:10.1016/j.cub.2021.04.022 (2021).

^124^ Schiffels, S. *et al.* Iron Age and Anglo-Saxon genomes from East England reveal British migration history. *Nat. Commun.* **7**, doi:10.1038/ncomms10408 (2016).

^125^ Seguin-Orlando, A. *et al.* Paleogenomics. Genomic structure in Europeans dating back at least 36,200 years. *Science* **346**, 1113–1118, doi:10.1126/science.aaa0114 (2014).

^126^ van de Loosdrecht, M. *et al.* Pleistocene North African genomes link Near Eastern and sub-Saharan African human populations. *Science* **360**, 548–552, doi:10.1126/science.aar8380 (2018)

^127^ Patterson, N., Price, A. L. & Reich, D. Population Structure and Eigenanalysis. *PLoS Genet* **2**, e190. https://doi.org/110.1371/journal.pgen.0020190 (2006)

^128^ Price, A. L. *et al.* Principal components analysis corrects for stratification in genome-wide association studies. *Nature Genetics* **38**, 904-909, doi:10.1038/ng1847 (2006).

^129^ Patterson, N. *et al.* Ancient admixture in human history. *Genetics* **192**, 1065-1093, doi:10.1534/genetics.112.145037 (2012).

^130^ The 1000 Genomes Project Consortium*.* A global reference for human genetic variation. *Nature* **526**, 68-74, doi:10.1038/nature15393 (2015).

^131^ Rubinacci, S., Ribeiro, D. M., Hofmeister, R. J. & Delaneau, O. Efficient phasing and imputation of low-coverage sequencing data using large reference panels. *Nature Genetics* **53**, 120-126, doi:10.1038/s41588-020-00756-0 (2021).

^132^ Walsh, S. *et al.* The HIrisPlex system for simultaneous prediction of hair and eye colour from DNA. *Forensic Sci Int Genet* **7**, 98-115, doi:10.1016/j.fsigen.2012.07.005 (2013).

^133^ Walsh, S. *et al.* Developmental validation of the HIrisPlex system: DNA-based eye and hair colour prediction for forensic and anthropological usage. *Forensic Sci Int Genet* **9**, 150-161, doi:10.1016/j.fsigen.2013.12.006 (2014).

^134^ Chaitanya, L. *et al.* The HIrisPlex-S system for eye, hair and skin colour prediction from DNA: Introduction and forensic developmental validation. *Forensic Sci Int Genet* **35**, 123-135, doi:10.1016/j.fsigen.2018.04.004 (2018).

^135^ Monroy Kuhn, J. M., Jakobsson, M. & Günther, T. Estimating genetic kin relationships in prehistoric populations. *PLoS One* **13**, e0195491, doi:10.1371/journal.pone.0195491 (2018).

^136^ Manichaikul, A. *et al.* Robust relationship inference in genome-wide association studies. *Bioinformatics* **26**, 2867–2873, doi:10.1093/bioinformatics/btq559 (2010)
