# Supplementary figures and images for: Bioarchaeology aids the cultural understanding of six characters in search of their agency (Tarquinia, ninth–seventh century BC, central Italy)
Source: Sci Rep. 2024 May 28;14:11895. doi: 10.1038/s41598-024-61052-z (PMC11133411; doi:10.1038/s41598-024-61052-z)

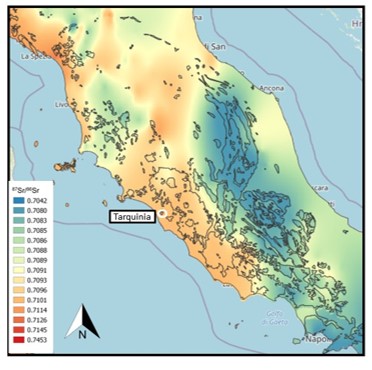

Supplement: Supplementary file 2 — Supplementary Figures. [file 41598_2024_61052_MOESM2_ESM.zip › Figure S1.jpg]

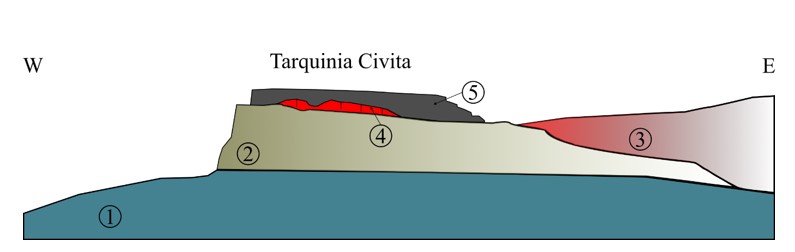

Supplement: Supplementary file 2 — Supplementary Figures. [file 41598_2024_61052_MOESM2_ESM.zip › Figure S2.jpg]

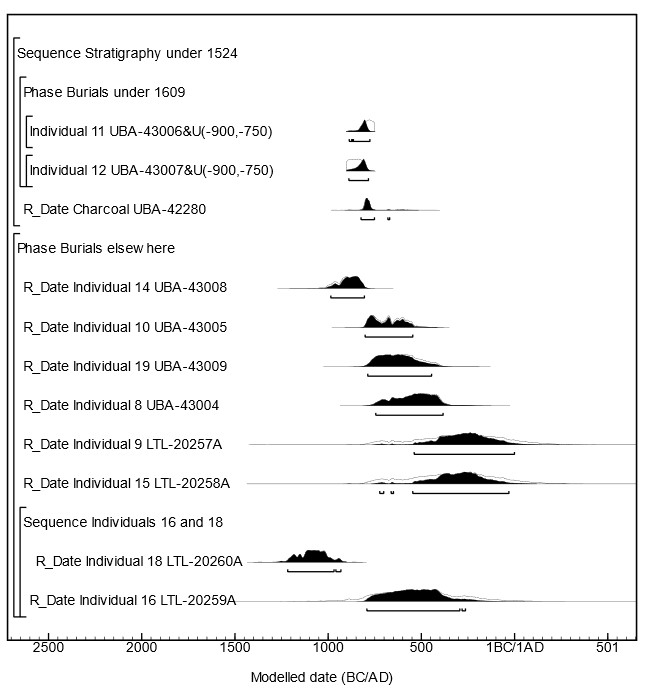

Supplement: Supplementary file 2 — Supplementary Figures. [file 41598_2024_61052_MOESM2_ESM.zip › Figure S3.jpg]

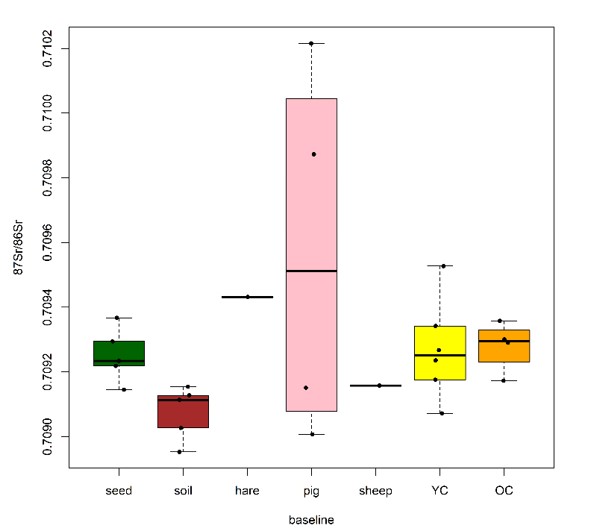

Supplement: Supplementary file 2 — Supplementary Figures. [file 41598_2024_61052_MOESM2_ESM.zip › Figure S4.jpg]
